# Dual RNA sequencing of group B *Streptococcus*-infected human monocytes reveals new insights into host–pathogen interactions and bacterial evasion of phagocytosis

**DOI:** 10.1038/s41598-023-28117-x

**Published:** 2023-02-06

**Authors:** Matthew J. Sullivan, Darren Prince, Kelvin G. K. Goh, Lahiru Katupitiya, Dean Gosling, Michael R. Crowley, David K. Crossman, Glen C. Ulett

**Affiliations:** 1 School of Pharmacy and Medical Sciences, and Menzies Health Institute Queensland, Griffith University, Gold Coast, QLD 4222 Australia; 2 The Department of Genetics, University of Alabama at Birmingham, 1720 Second Ave. S., Birmingham, AL 35294-0024 USA; 3 Present Address: School of Biological Sciences, University of East Anglia, Norwich, NR4 7TJ UK

**Keywords:** Bacterial pathogenesis, Bacteriology

## Abstract

*Streptococcus agalactiae*, also known as Group B *Streptococcus* (GBS) is a frequent cause of infections, including bacteraemia and other acute diseases in adults and immunocompromised individuals. We developed a novel system to study GBS within human monocytes to define the co-transcriptome of intracellular GBS (*iGBS*) and host cells simultaneously using dual RNA-sequencing (RNA-seq) to better define how this pathogen responds to host cells. Using human U937 monocytes and genome-sequenced GBS reference strain 874,391 in antibiotic protection assays we validated a system for dual-RNA seq based on measures of GBS and monocyte viability to ensure that the bacterial and host cell co-transcriptome reflected mainly intracellular (*iGBS*) rather than extracellular GBS. Elucidation of the co-transcriptome revealed 1119 dysregulated transcripts in *iGBS* with most genes, including several that encode virulence factors (*e.g., scpB*, *hvgA, ribD*, *pil2b*) exhibiting activation by upregulated expression. Infection with *iGBS* resulted in significant remodelling of the monocyte transcriptome, with 7587 transcripts differentially expressed including 7040 up-regulated and 547 down-regulated. qPCR confirmed that the most strongly activated genes included *sht*, encoding Streptococcal Histidine Triad Protein. An isogenic GBS mutant strain deficient in *sht* revealed a significant effect of this gene on phagocytosis of GBS and survival of the bacteria during systemic infection in mice. Identification of a novel contribution of *sht* to GBS virulence shows the co-transcriptome responses elucidated in GBS-infected monocytes help to shape the host–pathogen interaction and establish a role for *sht* in the response of the bacteria to phagocytic uptake. This study provides comprehension of concurrent transcriptional responses that occur in GBS and human monocytes that shape the host–pathogen interaction.

## Introduction

*Streptococcus agalactiae*, also known as Group B *Streptococcus* (GBS) is an opportunistic pathogen of humans and animals, which causes a diverse range of infections that encompasses asymptomatic colonization and severe and life-threatening systemic disease such as toxic shock-like syndrome^1^. GBS is the world’s foremost neonatal pathogen, causing significant morbidity and mortality in neonates following transmission from mother to baby during birth or resulting from neonates acquiring GBS after birth^2^. In addition to pneumonia and meningitis, GBS frequently causes bacteraemia^3^. In adults, GBS is frequently seeded into the bloodstream as a result of cellulitis or from urine^4^. Experimental systemic infection of mice shows that blood-borne GBS can also cause pyelonephritis secondary to high-level kidney infection^5^. However, while it is established that GBS frequently infects and can spread from the bloodstream, the interactions of this pathogen with phagocytic cells of the blood are not well understood. Within blood, GBS encounters monocytes and neutrophils; their activation, along with stimulation of non-phagocytic cells, can lead to production of cytokines and systemic hyper-inflammation^6^. In monocytes, GBS induces the production of multiple cytokines, with such responses in both neonatal and adult monocytes considered to support host defense^7^. However, an ability of GBS to subvert innate immune responses and evade phagocytosis enables the bacteria to prevent clearance from the bloodstream and contribute to processes that can ultimately lead to sepsis, as reviewed elsewhere^8^. In some severe cases, the bacteria can reach the blood–brain barrier and infect the central nervous system, as reviewed elsewhere^9^. Experimental models to study the complex interplay between GBS and human immune cells continue to emerge^8^.

One approach that is instrumental to understanding host–pathogen interactions and the interplay between bacteria and human cells is transcriptional analysis. The majority of studies using this approach have traditionally analysed transcripts of either the host or the pathogen, independently. For GBS, Mereghetti et al*.*^10^ revealed a rapid response of GBS NEM316 to contact with human blood, finding that GBS adapts to blood in a manner that is temperature dependant. Stitkiewicz and Musser^11^ explored transcriptional responses of GBS in growth media, finding that the GBS transcriptome is highly responsive to changing environments. Stitkiewicz et al*.*^11^ elucidated GBS transcriptional responses to amniotic fluid and identified dysregulated expression of multiple virulence factors. Finally, Shelver et al*.*^12^ exposed GBS to methionine in a study of bacterial adaptation to host environments, finding that MtaR is a regulator of gene expression that supports growth of the bacteria in plasma. Together, these studies based on transcriptional analysis underscore the value in analyzing GBS responses in defined conditions relevant to the host. However, few studies have examined transcriptional responses of GBS in situ in the context of a host niche such as inside infected host cells.

Technological developments in DNA sequencing and bioinformatics have laid the foundation for dual RNA sequencing (dual RNA-seq) with which the responses of a pathogen and host can be defined simultaneously^13–15^. This is valuable because analysis of the host and the pathogen concurrently using a single (infectious) sample provides new insight into the ways in which each organism adapts in situ to the context of the infectious process. Key dual RNA-seq studies have revealed intricate conversations between host and pathogen, for example, in bronchial epithelial cells infected with *Haemophilus influenza*^16^. Others have elucidated the temporal nature of macrophage responses to *Leishmania*^17,18^ and novel networks of host–pathogen interactions in mouse macrophages infected with *Candida albicans*^19^. A study of *Salmonella* showed the importance of sRNAs in host responses^13^. Thus, dual RNA-seq can help reveal important and new aspects of host–pathogen interactions. Notably, a dual RNA-seq analysis of Tilapia infected with GBS was reported recently^20^.

The aim of this study was to establish an approach suited to dual RNA-seq analysis to define the co-transcriptome of GBS-infected human monocytes. Additionally, we explored a targeted aspect of the dysregulated co-transcriptome of GBS using defined virulence assays to provide new insight into the host–pathogen interaction.

## Materials and methods

### Bacterial strains, plasmids and growth conditions

Strains of GBS and *E. coli* used in this study are listed along with plasmids in Supplementary Table S1. GBS was routinely grown in nutritive Todd-Hewitt Broth supplemented with 0.5% yeast extract (THY) or on THB agar (1.5% agar). *E. coli* was grown in Lysogeny Broth (LB) or on LB agar (1.5% agar). Media were supplemented with antibiotics appropriate for bacterial strains. For strains carrying plasmids, media was supplemented with Spectinomycin (Sp; 100 μg/mL) or Chloramphenicol (Cm; 10 μg/mL), as required. Complementation assays required supplementation with nisin from *Lactococcus lactis* (Sigma Aldrich #N5764 Lot 0000126560; 1.022 × 10^6^ IU/g) at ~ 20 IU/mL to induce *sht* expression.

### DNA extraction and genetic modification of GBS

Plasmid DNA from *E. coli* DH5α derivatives was routinely isolated using the QIAPrep spin plasmid miniprep kit (QIAGEN) according to the manufacturer’s instructions. GBS plasmid DNA was also isolated using this kit but with modification of the P1 buffer, which was supplemented with 30 mg/mL lysozyme and 100U of mutanolysin per 250 μL, and incubated for 1 h at 37 °C prior to the addition of the P2 buffer. Deletion of *sht* (CHF17_01323 / CHF17_RS06710 = ASZ01578.1) was constructed by allelic exchange using the temperature-sensitive pHY304aad9 plasmid as described previously^21,22^. Briefly, ~ 400–500 bp upstream and downstream of *sht* were amplified using primers (Supplementary Table S1) carrying 21–23 bp overlapping sequence complementary to the Cm cassette of pLZ12, to facilitate fusion of these amplicons to chloramphenicol acetyltransferase by 3-way PCR. The subsequent product was cloned into pHY304aad9, which was then electroporated into GBS 874391. Selection of transformants was performed as described previously^21^. A mutant carrying an in-frame, marked deletion in *sht* was generated by exploiting temperature selection of the plasmid, followed by loss of Sp resistance to identify a double cross-over mutant. Mutation in *sht* was validated by PCR using primers external to the mutation site and DNA sequencing. Complementation of *sht* was achieved *in trans* using a derivative of the *E. coli*-streptococcal shuttle vector pMSP3545^21,23^ using *Nco*I and *Spe*I sites to fuse *sht* to the ectopic promoter of *nisA*. Primers for PCR are shown in Table S1. Strains of GBS tagged with GFPmut3 and mCherry were used for microscopy and are described elsewhere^22,24^.

### Human monocytes

U937 human monocytes (ATCC CRL-1593.2) were purchased from ATCC. Cells were routinely grown in Roswell Park Memorial Institute (RPMI) 1640 medium (Life Technologies) supplemented with 2 mM L-glutamine as 1X GlutaMAX (Gibco), 1 mM sodium pyruvate (Gibco), 100 mM nonessential amino acids (Gibco), 100 U/mL penicillin, 100 mg/mL streptomycin (Gibco), 25 mM HEPES (Gibco), 10% heat-inactivated fetal bovine serum (FBS) (Gibco) and 25 µM β-mercaptoethanol (Gibco). For assays of monocytes with bacteria (including controls without either monocytes or bacteria), modified-RPMI (mRPMI) was prepared exactly as described above, but without supplementation with penicillin or streptomycin, and 1% FBS (instead of 10% FBS to limit growth of GBS). Monocytes were routinely counted using a NucleoCounter NC-200 and Via1-Cassette™ cartridges (Chemometec) to quantify viable cells.

### Antibiotic protection assay with GBS-infected monocytes

For assays that required sufficient mass of biological material for RNA isolation and sequencing, 24 mL of monocytes (approximately 3 × 10^6^ cells/mL) in mRPMI were infected with GBS that were prepared from overnight cultures in THY, washed in PBS and resuspended in mRPMI. A multiplicity of infection (MOI) of approximately 100 GBS per monocyte was used. Infected monocytes were centrifuged (5 min, 500 × *g*, RT) to enhance the interaction between GBS and monocytes (and increase yield of intracellular GBS from monocytes). Cells (24 mL total volume of monocytes + GBS) were split into 6 × 4 mL aliquots into the wells of 6-well cell culture plate and incubated at 37 °C with 5% CO_2_ for 1 h, at which time an antibiotic cocktail was added to kill extracellular GBS (final concentrations: 250 U/mL penicillin, streptomycin (Gibco), 50 µg/mL gentamicin (Sigma-Aldrich)). At 5 h post-antibiotic treatment, the cultures of infected monocytes (*iMon*) and *iGBS* for subsequent RNA extraction were sampled (1 mL) to quantify viable monocytes using Via1-Cassettes™ and Nucleocounter NC-200 (ChemoMetec) to stain cells with acridine orange and 4’,6-diamidino-2-phenylindole (DAPI) as a measure of live and dead cells, respectively. Intracellular GBS (*iGBS*) were quantified by retrospective colony counts by lysis of monocytes by brief exposure to PBS + 0.1% Triton-X-100 prior to serial dilution. The remainder (~ 23 mL) was harvested by centrifugation at 500 × *g* for 5 min. The pellet was resuspended in 1 mL PBS, pelleted at 500 × *g*, the supernatant discarded and cell pellet was snap frozen and stored at − 80 °C for later use. GBS cultures prepared in the absence of monocytes were used as controls for transcriptional responses detected in intracellular GBS. Control GBS (*cGBS*) were grown in duplicate wells of 6-well cell culture plates (8 mL total volume) at 37 °C with 5% CO_2_ for 6 h in mRPMI as above. At harvest, cells were centrifuged at 13,000 × *g*, washed once in 1 mL PBS and cell pellets (approximately 2 × 10^8^ CFU) were snap frozen for subsequent RNA extraction as above. Non-infected monocytes were prepared in the absence of GBS and used as controls for the responses of monocytes detected following infection with GBS. Control monocyte (*cMon*) cultures (approximately 3 × 10^6^ cells/mL) were prepared in 8 mL volume and split into duplicate wells of 6-well cell culture plates at 37 °C with 5% CO_2_ for 1 h at which time the same antibiotic cocktail as described above was added. Then, at 5 h post-antibiotic treatment, the monocytes were enumerated, snap frozen and stored at − 80 °C as cell pellets for subsequent RNA extraction as above. For all groups (*iMon/iGBS*; *cGBS*; *cMon*) at least 4 independent RNA isolations were obtained for downstream qPCR or sequencing.

For assays to quantify and compare viable *iGBS* from infected monocytes and WT 874391 or Δ*sht* (GU2843) strains, identical cell densities of monocytes (~ 3 × 10^6^ cells/mL) and bacteria (~ 3 × 10^8^ GBS/mL) were used and performed essentially as described above, but in smaller volumes, as follows. Monocytes were prepared at 3.3 × 10^6^ cells/mL in mRPMI and 900 μL was added to individual wells of a 24 well plate (Costar 3738, Corning). Bacteria from overnight cultures in THY were washed in PBS and prepared in mRPMI at a concentration of 3 × 10^9^ CFU/mL; 100 μL was inoculated into the wells of 24-well plates containing monocytes (1 plate used per timepoint). Infected monocytes in 24-well plates were centrifuged (5 min, 500 × *g*, RT) to enhance the interaction between GBS and monocytes (as above). For T = 0 h controls, plates were processed immediately after centrifugation for CFU/mL counts (below). After centrifugation, 24-well plates were incubated at 37 °C with 5% CO_2_ for 1 h, at which time 30 μL of an antibiotic cocktail was added to kill extracellular GBS (final concentrations: 250U/mL penicillin, streptomycin (Gibco), 50 µg/mL gentamicin (Sigma-Aldrich)). After incubation with antibiotics, samples were processed for CFU/mL counts as follows. Each 1 mL volume was centrifuged at (5 min, 500 × *g*, RT) in 1.5 mL microfuge tubes and 5 washes were performed by gently discarding supernatant and resuspending pellets in PBS. After washes, *iGBS* were quantified by retrospective colony counts following lysis of monocytes by resuspension and brief exposure (2 min) to PBS + 0.1% Triton-X-100 prior to serial dilution and plating onto agar. For assays that incorporated the Δ*sht* complemented strain (GU3178), we also included empty vector controls for WT (GU3181) and Δ*sht* (GU3180) backgrounds (Δ*sht*^#^ and WT^#^, respectively); bacterial starter cultures and mRPMI were supplemented with Sp and nisin (20 IU/mL) for plasmid maintenance and induction of *sht* from pGU3173; otherwise, identical conditions and cell densities were used in 1 mL assay volumes using 24-well plates.

### RNA extraction and analysis

Cell pellets (comprising infected monocytes) were resuspended in 500 µL RNase-free TE Buffer containing 30 mg/ml lysozyme in 2 mL nuclease-free, cap-lock tubes (Eppendorf) containing 50 mg of pre-sterilized acid-washed glass beads (425–600 µm (Sigma)). The suspensions were subject to bead-beating (10 s at frequency 30) using a Tissue Lyser II (QIAGEN) to release *iGBS* from the monocytes to aid enzymatic digestion of the bacterial cells. Then, 50 µl (500 U) of mutanolysin was added prior to incubation for 90 min at 37 °C. Following digests, cell mixtures were transferred 15 mL tubes and subjected to RNA isolation using the SV Total RNA isolation system (Promega) with some modifications. Lysis buffer was added at a ratio of 175 µl per 5 × 10^6^ viable monocytes in the initial cell pellet. The mixture was passed through a 20 gauge needle ten times to shear genomic DNA. Twice the volume of RNA dilution buffer (to lysis buffer) was added and the mixture was inverted before aliquoting into four 2 mL tubes for incubation (3 min at 70 °C). After centrifuging (10 min at 13,000 × *g*, 4 °C), supernatants were combined into a 15 mL tube and cold ethanol was added at a ratio of 200 µL per 500 µL supernatant. The supernatants were aliquoted into ten spin columns; the remainder of the RNA isolation protocol was performed according to the manufacturer’s instructions, including the on-column DNase treatment. RNA was eluted using nuclease-free Ultrapure Distilled Water (volumes shown in Supplementary Table S2). Trace DNA contamination was removed by an additional DNase treatment step (Turbo DNAfree kit from Ambion), the success of which was confirmed by PCR. GBS DNA contamination was tested by PCR for the *spb1* gene to generate a 762 bp amplicon. The PCR was performed using Phusion High-Fidelity DNA polymerase (Thermo-Scientific) following the manufacture’s protocol with *spb1*-specific primers 1279-F1 ATTGCGACATGGGCTAAATC and 1279-R1 TGAAGCTTTTGTGGAACCATG. Monocyte DNA contamination was tested by PCR for the *gapdh* gene to generate a 171 bp amplicon. The *gapdh*-specific primers were Human_GAPDH-DWP-F7 GGAAATGAGCTTGACAAAGTGG and Human_GAPDH-DWP-R7a GAGCACAGGGTACTTTATTGATGG. RNA quality was analysed using Experion Eukaryotic and Prokaryotic RNA StdSens Reagents (BIO-RAD) and RNA StdSens Chips (BIO-RAD) in an Experion Automated Electrophoresis Station (BIO RAD). Electropherograms and virtual gel images used in Supplementary Fig. S1 were generated using reports from BIO-RAD Experion™ software.

### cDNA synthesis and qPCR

RNA samples from independently prepared cultures of infected monocytes were used to prepare cDNA for RNA-seq (n = 4) as well as qRT-PCR (n = 4). cDNA synthesis was performed using SuperScript III Reverse Transcriptase kits (Life Technologies), according to the manufacturer’s instructions. Amounts of RNA used for each group are shown in Supplementary Table S2. Primers for qRT-PCR are listed in Supplementary Table S1 and were designed using Primer3 Plus^25,26^.

qPCR was performed in 384-well plates on a QuantStudio 6-Flex Real-Time PCR system. Reactions were performed in triplicate or quadruplicate for each condition in accordance with MIQE guidelines^27^. Each 10 µL reaction consisted of 5 µL (1X) 2X SensiFAST SYBR No-Rox Mix (Bioline), 0.2 µL (400 nM) each of 20 µM forward and reverse primers, 1 µL of template, and 3.6 µL of water. Amplification was 1 cycle at 95 °C for 2 min, followed by 40 cycles alternating between 95 °C for 5 s and 60 °C for 30 s. Reactions used 1:160 dilutions in water of all cDNA templates. We analyzed 14 bacterial and 14 human genes, including housekeeper genes for each (GBS: *dnaN*, monocytes; *gapdh*, *β-actin*). For optimization of reaction conditions for each primer pair, we used genomic DNA from GBS (8 ng) and from infected monocytes (28 ng). Data analysis was performed using the QuantStudio Real-Time PCR Software (v.1.1). Standard curves were generated using five point serial dilutions of genomic DNA (fivefold) from GBS 874391^28^ and used to calculate primer efficiencies and relative transcript amounts. Relative expression ratios (fold-change) were calculated using C_T_ values and primer efficiencies as described using the method of Pfaffl^29^. Relative mRNA transcript amounts or expression ratios were normalized using *dnaN*, encoding DNA polymerase III β-subunit, from four independent biological replicates.

### Dual RNA-seq and bioinformatics

In preparation for Dual RNA-seq, we performed whole-genome sequencing of GBS strain 874391 using long (PacBio) and short (Illumina) read sequencing, respectively, and published this elsewhere^28^. The complete, closed genome of strain 874391 is available from GenBank under accession CP022537. Cultures were prepared as described above for *RNA extraction and analysis*. RNase-free DNase-treated RNA that passed Bioanalyzer 2100 (Agilent) analysis was used for RNA sequencing (RNA-seq) using the Illumina NextSeq 500 platform. We used the NEB Next Ultra II directional RNASeq kit for library preparation. The RNA samples containing both bacterial and human RNA were subjected to ribosome reduction including probes for both species. The cDNA libraries were quantitated using qPCR in a Roche LightCycler 480 with the Kapa Biosystems kit (Kapa Biosystems, Woburn, Massachusetts) prior to cluster generation. We ran paired-end 2 × 75–bp sequencing runs to align the cDNA sequences to the reference genome (or single-end for bacteria-only samples). Mixed RNA samples containing human RNA and bacterial RNA were processed similarly but enrichment for prokaryotic RNA was performed to account for low ratios of abundance of bacterial:human RNA. For data preprocessing and bioinformatics, STAR (version 2.7.3a) was used to align the raw RNA-seq fastq reads to the GBS 874391 reference genome^28^, and the human reference genome GRCh38 p13 Release 32 from Gencode. HTSeq-count, version 0.11.1, was used to estimate transcript abundances^30^. DESeq2 was then used to normalized and test for differential expression and regulation. Genes that met certain criteria (i.e. fold change of >  ± 2.0, q value of < 0.05 were accepted as significantly altered in expression^31^. Raw and processed data were deposited in Gene Expression Omnibus (accession no. GSE161013). InnateDB^32^ was used to analyse Gene Ontology (GO) Overrepresentation Analysis (ORA) of the Biological Processes in *iMon* based on RNA-seq data. The input was a list of fold-change and P-adj values of the 7587 differentially expressed genes. The GO term ORA P-values were generated using the Hypergeometric Distribution test of whether a pathway is statistically more over-represented in the dataset than by chance. P-adj are P-values corrected for multiple testing by the Benjamini and Hochberg method^33^.

### Microscopy

Fifty thousand monocytes were infected as described above with GFPmut3-GBS^24^ or mCherry-GBS^22^ for 1 h, followed by application of antibiotics as described above. Then, 5 h later, the infected cells were subjected to three washes of PBS and fixed for 15 min at 37 °C using 3.5% (wt/vol) paraformaldehyde. The cells were concentrated by centrifugation and mounted using ProLong™ Glass antifade mounting medium (Invitrogen). The cells were visualized using a Zeiss AxioImager.M2 microscope (Carl Zeiss MicroImaging) fitted with a Plan-Apochromat X63/1.40 lens objective and AxioCam MRm Rev.3 and MRc 5 cameras. Images of cells were captured with 63HE and 44 filter sets (to detect mCherry [587 nm, 610 nm], GFPmut3 [494 nm, 518 nm] fluorescence, respectively, with excitation and emission spectra listed consecutively for each) and phase contrast with Zen Pro (version 2) software. Composite images were captured as a collection of 20–26 Z-stacked micrographs covering a total depth of approximately 8–10 μm, which were compiled using maximum intensity projection to generate two-dimensional images.

### Mouse infection assays

C57BL/6 mice were purchased from the Animal Resources Centre (Canning Vale, WA) and were at 6–8 weeks of age at the time of inoculation. Experiments were performed using a 50:50 male to female ratio. Mice were challenged intravenously (i.v.) with 1 × 10^7^ CFU of GBS 874391 or mutant derivatives in 200 µL PBS. Mice were housed in groups of five. For the collection of tissues, mice were euthanized by isoflurane anesthesia overdose followed by cervical dislocation. Blood was collected by cardiac puncture and used for colony counts on Trypticase soy agar (TSA) with 5% horse blood. Brain, heart, lungs, liver, kidneys, spleen and bladder tissues were weighed, homogenized and diluted in PBS for colony counts per mg tissue. Independent experiments were repeated two times.

### Ethics

This study was carried out in accordance with the guidelines of the Australian National Health and Medical Research Council. The Griffith University Animal Ethics Committee reviewed and approved all experimental protocols for animal usage according to the guidelines of the National Health and Medical Research Council (Approval: MSC/01/18/AEC). The study is reported in accordance with ARRIVE guidelines.

### Statistical methods

Comparisons of data used Mann–Whitney *U* tests, unpaired *t-*tests, one-way ANOVA or Kruskall-Wallis tests followed by post-hoc multiple comparisons tests as described in the Figure legends. The description of *n* denotes the number of biological replicates derived from independent experiments (bars and error bars in Figures represent Mean and Standard Error of the Mean (SEM). Statistical significance was accepted with P values of ≤ 0.05. All data were analysed using GraphPad Prism V8.

## Results

### Optimizing antibiotic protection assay, antibiotic time-kill curve, and recovery of viable GBS

**Figure 1:**
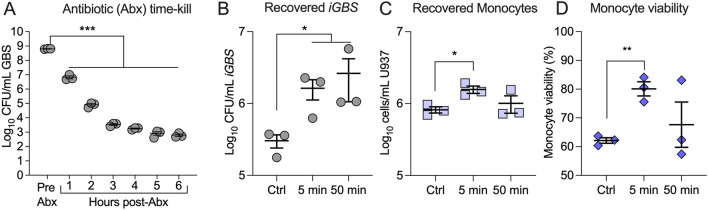
Recovery and viability of GBS and monocytes from antibiotic protection assay developed for co-transcriptome analysis. Number estimates of viable GBS recovered from RPMI containing 1% FBS and other supplements, including antibiotics (250U/mL penicillin, 250U/mL streptomycin, and 50 µg/mL gentamicin) according to CFU assays were performed over a time-kill course post-addition of antibiotics (Abx) (**A**). Recovery of viable intracellular GBS (*iGBS*) from infected monocytes using protocols of no centrifugation as control (Ctrl), or centrifugation (500 × *g*, RT) for 5 min, or 50 min (**B**). The number (**C**) and viability (**D**) of monocytes recovered at 6 h post-infection, comparing Ctrl conditions without centrifugation and centrifugation for 5 min, or 50 min. Bars show means and SEMs representive of 4 independent experiments and analysed using One-way ANOVA with Holm Sidak Multiple comparisons. (*P ≤ 0.05; **P ≤ 0.01; ***P ≤ 0.001).

Extensive optimizations were performed initially to validate the in vitro antibiotic protection assay conditions that would ensure sufficient yield and quality of RNA from viable intracellular GBS (*iGBS*) that were to be recovered from infected monocytes. Initially, we defined the conditions for antibiotic treatment of infected monocytes, which would maximise recovery of viable *iGBS* from infected monocytes but minimize recovery of viable GBS that were extracellular to monocytes meaning that the recovered bacterial RNA for downstream sequencing would be principally from *iGBS* (not extracellular GBS). Other assay conditions, including the duration of incubation, growth medium, scale of volumes and protocols for centrifugation and for cell collection and RNA isolation (see “Materials and Methods”) were also optimized to ensure suitable RNA recovery for robust dual RNA-seq. For the antibiotic aspect, time-kill curves for GBS exposed to the antibiotic cocktail in the culture medium (250 U/mL penicillin, 250 U/mL streptomycin and 50 µg/mL gentamicin) in the absence of monocytes demonstrated efficient reduction in the viability of (extracellular) GBS after 5 h of incubation according to CFU estimates; these conditions reduced the number of viable GBS recovered from the media by several orders of magnitude after 5 h in the absence of monocytes (Fig. 1A).

In monocytes that were exposed to GBS for 1 h (MOI approximately 100: 1; bacteria: monocyte) and centrifuged briefly (5 min) to enhance the interaction between GBS and host cells, then subsequently treated with antibiotics for 5 h, > 10^6^ CFU were routinely recovered from monocytes (Fig. 1B); in these assay conditions, this degree of recovery equates to a > 99.99% recovery of *iGBS* (at > 10^6^ CFU/mL) because the antibiotic conditions used for 5 h kills the vast majority of GBS that would be external to the monocytes (compare 10^6^–10^7^ CFU/mL at 1 h and 10^2–3^ CFU/mL at 5 h in Fig. 1A, equating to a reduction in the numbers of viable GBS of > 99.99% in the extracellular environment). The yield of viable bacteria was substantially lower in the absence of a centrifugation step, or with a duration of centrifugation longer than 50 min.

The number and viability of infected monocytes at 6 h post-exposure to GBS was > 10^6^ cells/mL and approximately 80%, respectively (Fig. 1C,D). We accepted these conditions as suitable to generate mixed RNA populations from co-cultures of GBS-infected monocytes, validated as bacterial RNA principally from *iGBS* not extracellular GBS in addition to RNA from the infected monocytes.

### Analysis of co-transcriptome of *iGBS* and human monocytes

Initially, we analyzed the prokaryotic-eukaryotic mixed RNA population from infected monocytes containing *iGBS* using PCR to test for contaminating genomic DNA (gDNA) from monocytes or GBS (and exclude positive samples). Using GBS-specific *spb1*-targeting and human *gapdh*-targeting primers, PCR showed that an additional DNase treatment step was essential to remove gDNA and ensure the mixed RNA populations were free of gDNA (Supplementary Fig. S1). Analysis of RNA from mixed population RNA from infected monocytes containing *iGBS* (Supplementary Fig. S1B) confirmed suitable RNA quality and quantity (details in Supplementary Table S2) for cDNA synthesis and sequencing. Controls included RNA from independent pure cultures of GBS without monocytes in equivalent media and conditions, and non-infected monocytes, which were analyzed using equivalent approaches prior to use in dual RNA-seq.

**Figure 2:**
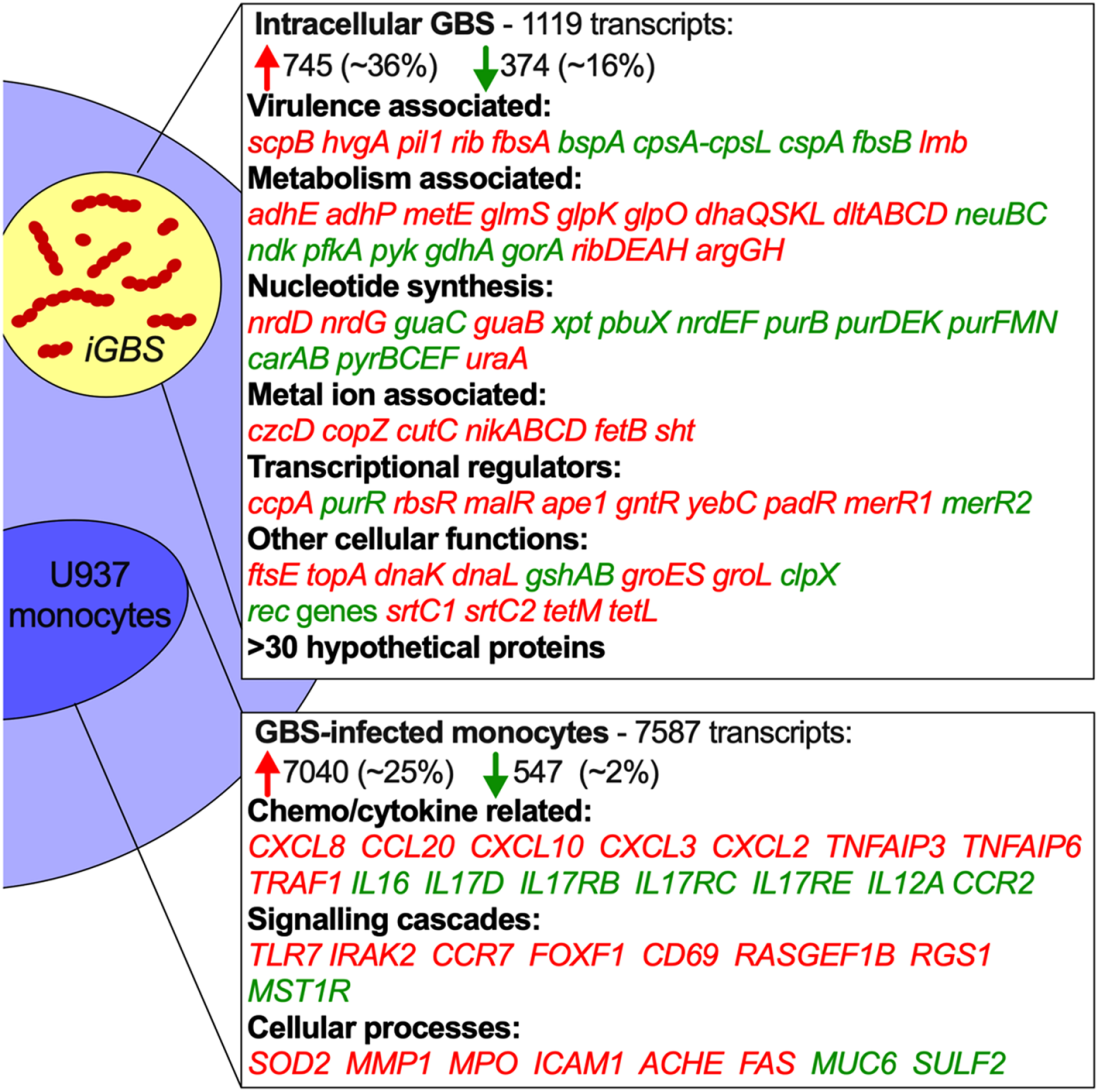
The co-transcriptome of GBS-infected human monocytes. Extensive reshaping of transcriptional activity in human monocytes containing *iGBS* (6 h infection) highlighting a selection of strongly activated genes with upregulated transcription (red) or downregulated transcription (green) during the host–pathogen interaction. A total of 1119 dysregulated transcripts were detected in *iGBS* with 745 upregulated (red) and 374 downregulated (green) transcripts (± twofold; P-adj < 0.05). Percentages of transcripts upregulated or downregulated were calculated using total numbers of mapped transcripts for GBS (2,100; using accession NZ_CP022537.1) and U937 monocytes (28,516; using accession hg38(GRCh38.p13)). These numbers for GBS were: total number of transcripts upregulated = 745/2100 (35.5%) compared to downregulated = 374/2100 (17.8%), and unchanged = 981/2100 (46.7%). The equivalent numbers for monocytes were: total number of transcripts upregulated = 547/28,516 (1.9%) compared to downregulated = 7040/28,516 (24.7%), and unchanged = 20,929/28,516 (73.4%).

**Figure 3:**
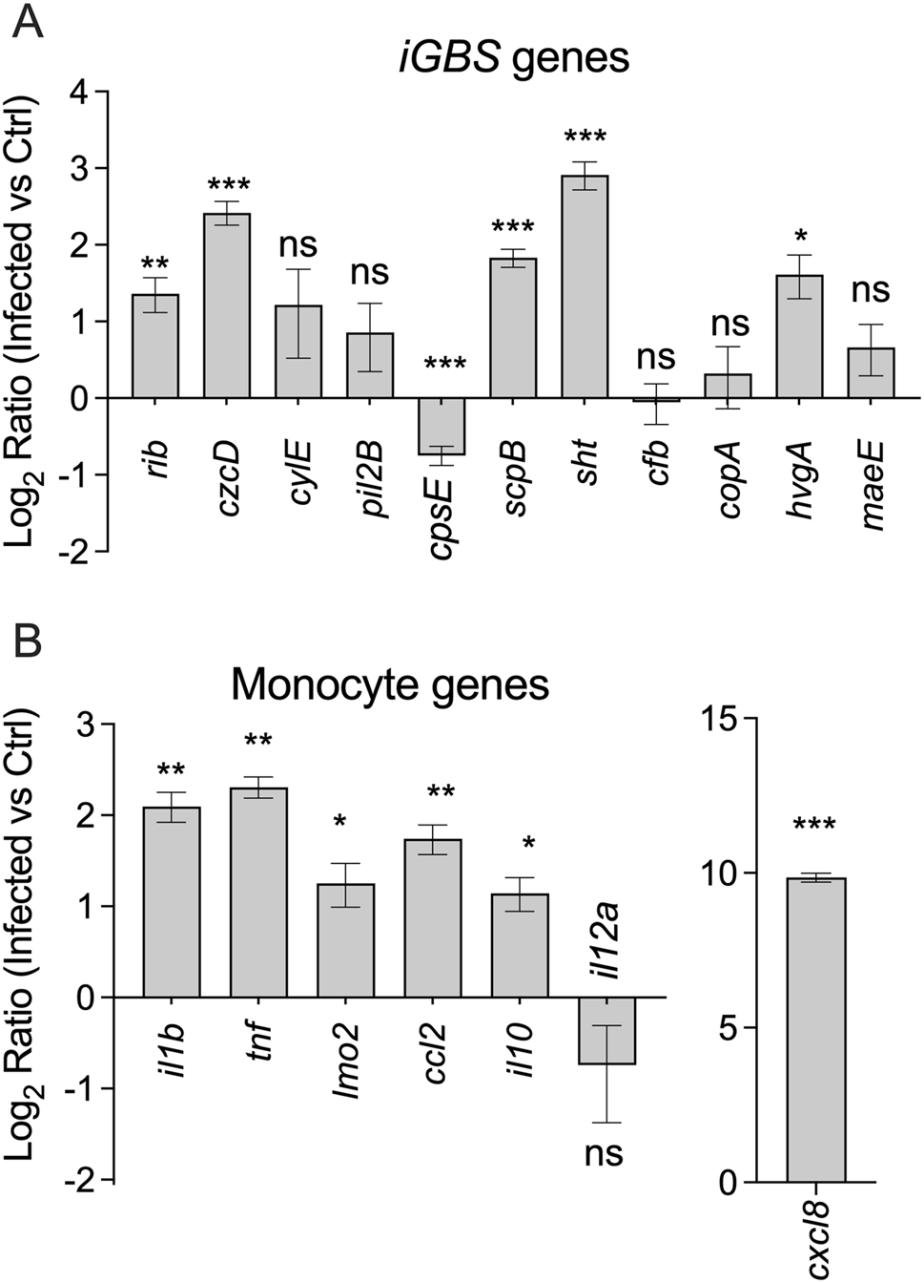
qPCR validation data for genes of the monocyte co-transcriptional response. Primers targeting *iGBS* (**A**) or monocyte transcripts (**B**) were used in qRT-PCR assays comparing expression in infected vs control conditions. Ratios in A were calculated as described previously^29^ using CT values, primer efficiencies and housekeeping *dnaN* for *iGBS* and *gapdh* for human monocytes. Ratios were compared using One sample t-tests vs theoretical mean Ratio of 1 (*P ≤ 0.05; **P ≤ 0.01; ***P ≤ 0.001).

Co-transcriptional analysis of *iGBS* and infected monocytes at 6 h after infection showed extensive and simultaneous reshaping of the transcriptional landscape in pathogen and host (Fig. 2). We detected 1119 total dysregulated transcripts in *iGBS* with 745 upregulated and 374 downregulated transcripts (± twofold, P-adj < 0.05; Fig. 2). These included genes encoding virulence factors *scpB*, *hvgA*, *pil1*, *rib*, *fbsA*, *bspA*, *cpsA-cpsL-neuB-neuC* and *cspA*. The 50 most significantly differentially expressed genes in *iGBS* are summarized in Table 1 and Supplementary Dataset S1. We noted expression of the hemolysin-associated genes *cylXDGacp* but no change in *cylE* that is central in the synthesis pathway for the GBS ornithine rhamnolipid hemolysin. There were numerous genes associated with aspects of cellular metabolism (*e.g., adhE*, *adhP*, *metE*, *gdhA*, *ribDEAH* and *argGH*), de novo purine, pyrimidine and deoxynucleotide synthesis (*e.g., nrdDG*, *nrdEF*, *guaB*, *guaC*, *pyr* genes, *pur* genes, *carAB*) and genes associated with metal ion homeostasis with *iGBS* engaging numerous Cu- and Zn-responsive genes (*e.g., czcD*, *copZ*, *cutC*, *nikABCD* and *sht*). In addition, genes for several transcriptional regulators (*e.g., ccpA*, *purR*, *rbsR*, and *malR*) and other cellular processes (*e.g., ftsE*, *topA*, *dnaK*, *gshAB*, *groES*, and *srtC1/2*). qPCR analysis of several genes that were dysregulated in *iGBS* showed a consistency of findings with the dual RNA-seq data (Fig. 3A). Specifically, analysis of twelve genes showed three (*pil2B*, *cfb*, *copA*) with no significant changes in expression in *iGBS* compared to bacteria-only controls; several other genes, including *cpsE*, *sht*, *czcD*, *scpB* and *hvgA* exhibited significant changes in expression comparing *iGBS* to bacteria-only controls, consistent with and confirming the findings of dual RNA-seq.

**Table 1:** Top transcriptional activation (upregulated) and repression (downregulated) responses of *iGBS* in monocytes.

Gene locus tag	Label	Gene product	FC	P-adj
CHF17_RS00245		tRNA-Ile	232.25	2.17E − 11
CHF17_RS08755		PTS beta-glucoside transporter subunit IIBCA	198.45	0.00E + 00
CHF17_RS03815		tetracycline resistance determinant leader peptide	197.68	7.30E − 26
CHF17_RS00445	*adhP*	Alcohol dehydrogenase AdhP	173.27	0.00E + 00
CHF17_RS01525	*lrgB*	Antiholin-like protein LrgB	157.58	1.03E − 281
CHF17_RS08465		zn-dependent alcohol dehydrogenase family protein	104.75	1.19E − 254
CHF17_RS08750		ROK family protein	103.56	0.00E + 00
CHF17_RS01520		CidA/LrgA family protein	97.13	5.08E − 75
CHF17_RS02320		23S ribosomal RNA	87.20	2.56E − 08
CHF17_RS02745		tRNA-Ile	66.90	1.32E − 05
CHF17_RS00240		tRNA-Gly	57.83	2.44E − 19
CHF17_RS00440	*adhE*	Bifunctional acetaldehyde-CoA/alcohol dehydrogenase	57.11	2.35E − 158
CHF17_RS01970	*glpK*	Glycerol kinase GlpK	56.34	2.18E − 65
CHF17_RS08545	*dhaS*	Dihydroxyacetone kinase transcriptional activator	51.87	6.33E − 229
CHF17_RS04030		ABC-2 family transporter protein	50.06	0.00E + 00
CHF17_RS01980		Aquaporin family protein	48.84	1.86E − 208
CHF17_RS01975	*glpO*	Type 1 glycerol-3-phosphate oxidase	45.01	4.08E − 56
CHF17_RS05665		L,D-transpeptidase	44.41	1.67E − 140
CHF17_RS01560	*treP*	PTS system trehalose-specific EIIBC component	38.09	4.87E − 250
CHF17_RS05275		CatB-related O-acetyltransferase	34.53	2.24E − 180
CHF17_RS07525		30S ribosomal protein S21	33.65	1.37E − 64
CHF17_RS03425	*rplS*	50S ribosomal protein L19	32.43	5.52E − 281
CHF17_RS04020		HYPOTHETICAL protein	32.07	0.00E + 00
CHF17_RS04025		ABC-2 family transporter protein	31.80	6.61E − 186
CHF17_RS10785		DUF1304 domain-containing protein	29.84	1.17E − 37
CHF17_RS08890		Helix-turn-helix transcriptional regulator	− 15.17	3.80E − 188
CHF17_RS00770		Helix-turn-helix transcriptional regulator	− 15.96	2.58E − 191
CHF17_RS01005		1,4-beta-N-acetylmuramidase	− 16.26	5.61E − 115
CHF17_RS06625		Hypothetical protein	− 16.44	3.74E − 235
CHF17_RS00720		Hypothetical protein	− 16.85	6.73E − 158
CHF17_RS00765		Hypothetical protein	− 18.75	1.55E − 253
CHF17_RS00760		Hypothetical protein	− 20.53	0.00E + 00
CHF17_RS00750		Hypothetical protein	− 22.04	8.00E − 237
CHF17_RS00965		Phage tail tape measure protein	− 22.09	1.80E − 171
CHF17_RS00745		ImmA/IrrE family metallo-endopeptidase	− 25.08	2.11E − 251
CHF17_RS00985		DUF1366 domain-containing protein	− 25.25	1.43E − 36
CHF17_RS00980		Hypothetical protein	− 26.72	4.26E − 237
CHF17_RS00780		ORF6C domain-containing protein	− 27.32	0.00E + 00
CHF17_RS11305		Hypothetical protein	− 31.40	6.73E − 284
CHF17_RS00920		Phage portal protein	− 41.99	1.29E − 248
CHF17_RS00925		Clp protease ClpP	− 42.84	2.80E − 156
CHF17_RS00935		Phage head–tail connector protein	− 45.16	1.39E − 107
CHF17_RS00930		Phage major capsid protein	− 48.66	0.00E + 00
CHF17_RS00775		Hypothetical protein	− 55.68	0.00E + 00
CHF17_RS00960		Hypothetical protein	− 58.80	1.16E − 31
CHF17_RS00940		Head–tail adaptor protein	− 66.69	2.28E − 69
CHF17_RS00955		Hypothetical protein	− 100.50	4.67E − 172
CHF17_RS11320		Hypothetical protein	− 129.22	4.41E − 63
CHF17_RS00945		HK97 gp10 family phage protein	− 215.30	9.57E − 31
CHF17_RS00950		HYPOTHETICAL protein	− 386.98	1.57E − 75

In infected monocytes, we detected a dramatic transcriptional signature encompassing genes that encode innate immune response factors, cytokines and host defence molecules in response to GBS. Monocyte genes that were significantly activated (± twofold, P-adj < 0.05) included *IL8*, *IL1alpha*, *IL1beta*, *TNF*, *MCP-1* (Fig. 2). The most strongly activated genes were *IL8* (*CXCL8*) and *CXCL10* (also known as Interferon gamma-induced protein 10), which exhibited 94 and 117-fold increases in expression compared to monocyte-only controls, according to dual RNA-seq. We used qPCR to analyze 12 host-related genes, including those with no significant fold change (*IL12A*), modest (e.g. *TNF*, *LMO1, IL10*, *CCL2*, *IL1B*) or high fold change, including CXCL8 which was among the 50 identified to be most significantly dysregulated in monocytes as a result of infection (Table 2 and Supplementary Dataset S1). Seven of the twelve genes were shown to be significantly upregulated in infected monocytes by more than two-fold compared to monocyte-only controls (*IL8*, *IL1alpha*, *IL1beta*, *TNF*, *IL10*, *MCP-1*, *LMO2*), according to qPCR. The remaining four genes (*IL6*, *IL12b*, *IL17*, *CXCL1*) yielded insufficient amplification data for meaningful analysis (even at 45 cycles of PCR). These results showed a consistency in detection of significant transcriptional responses for multiple genes comparing to qPCR to dual RNA-seq, including for *IL1beta*, *TNF*, and *CCL2* (Fig. 3B). The most significant Biological Processes (generated by Gene Ontology analysis within innateDB^34^ and ranked according to significance from overrepresentation analysis [ORA]) are summarized in Supplementary Table S3. These data highlight the extensive activation of numerous processes including immune responses, innate immune responses, inflammatory responses, responses to lipopolysaccharide, chemotaxis and cell cycle arrest. Deactivation of processes included RNA processing, gene expression, DNA replication, ATP catabolic processes, mRNA splicing via spliceosome and ferrous iron transport. Taken together, these findings reveal that human monocytes generate a dramatic co-transcriptional response after 6 h of infection with GBS characterized by extensive cytokine and innate immune processes that are associated with host defence.

**Table 2:** Top transcriptional activation (upregulated) and repression (downregulated) responses of human monocytes infected with GBS.

ENSEMBL ID*	Label	Gene product	FC	P-adj
00000169245.6	CXCL10	C-X-C motif chemokine ligand 10	117.7	7.4E − 43
00000169429.11	CXCL8	C-X-C motif chemokine ligand 8	94.4	2.4E − 49
00000243455.3	RPS18P13	Ribosomal protein S18 pseudogene 13	89.7	6.2E − 08
00000123610.5	TNFAIP6	TNF alpha induced protein 6	61.8	1.3E − 09
00000270390.1	SFXN4P1	SFXN4 pseudogene 1	48.5	5.6E − 11
00000225286.1		Ribosomal protein S2 (RPS2) pseudogene	44.3	2.0E − 04
00000118503.15	TNFAIP3	TNF alpha induced protein 3	42.1	2.9E − 225
00000115009.13	CCL20	C–C motif chemokine ligand 20	36.4	3.7E − 28
00000138670.17	RASGEF1B	RasGEF domain family member 1B	33.5	2.7E − 07
00000090104.12	RGS1	Regulator of G protein signaling 1	33.3	5.3E − 51
00000250033.5	SLC7A11-AS1	SLC7A11 antisense RNA 1	26.9	2.5E − 47
00000224969.1		Novel transcript	25.4	8.7E − 03
00000269540.1		Novel zinc finger protein pseudogene	22.9	7.0E − 04
00000163734.4	CXCL3	C-X-C motif chemokine ligand 3	22.5	9.3E − 04
00000235763.1	SNRPGP5	Small nuclear ribonucleoprotein polypeptide G^#^ 5	21.3	1.9E − 02
00000173237.4	C11orf86	Chromosome 11 open reading frame 86	19.9	7.2E − 03
00000168367.11	LINC00917	Long intergenic non-protein coding RNA 917	19.6	7.6E − 03
00000198576.4	ARC	Activity regulated cytoskeleton associated	19.6	2.8E − 02
00000262400.1		Calcium activated nucleotidase 1 (CANT1)^#^	17.7	4.2E − 02
00000081041.9	CXCL2	C-X-C motif chemokine ligand 2	16.7	6.7E − 36
00000196664.5	TLR7	Toll like receptor 7	16.6	1.1E − 03
00000105246.6	EBI3	Epstein-Barr virus induced 3	16.0	1.0E − 10
00000265237.1	MIR3142	microRNA 3142	16.0	2.0E − 02
00000260542.1		Novel transcript	15.7	7.6E − 04
00000196611.5	MMP1	Matrix metallopeptidase 1	15.3	6.2E − 17
00000286274.1		Novel transcript	− 46.3	6.6E − 03
00000229204.3	PTGES3P3	Prostaglandin E synthase 3 pseudogene 3	− 46.5	5.1E − 03
00000185155.11	MIXL1	Mix paired-like homeobox	− 46.7	1.0E − 04
00000238205.3	MPC1L	Mitochondrial pyruvate carrier 1 like	− 47.0	1.1E − 02
00000271379.1		Family with sequence similarity 173, member B^#^	− 47.7	6.9E − 04
00000196562.14	SULF2	Sulfatase 2	− 48.9	2.4E − 12
00000163106.11	HPGDS	Hematopoietic prostaglandin D synthase	− 49.0	4.5E − 03
00000270811.1		Ribosomal protein L21 (RPL21) pseudogene	− 49.1	8.9E − 03
00000173250.2	GPR151	G protein-coupled receptor 151	− 51.7	1.8E − 14
00000263096.1		Novel transcript, antisense to HLF	− 52.0	2.5E − 03
00000257065.1		Novel protein	− 53.5	2.6E − 03
00000223882.1	ABCC5-AS1	ABCC5 antisense RNA 1	− 54.5	2.7E − 05
00000259362.2		Novel transcript	− 55.3	2.5E − 03
00000214070.3	MANEALP1	MANEAL pseudogene 1	− 55.3	2.2E − 03
00000223375.1		Ribosomal protein S26 (RPS26) pseudogenes	− 55.6	4.5E − 03
00000269681.1		CEACAM18^#^^	− 60.8	1.1E − 03
00000281048.2		Novel transcript, antisense to PROP1	− 60.8	1.1E − 03
00000146477.6	SLC22A3	Solute carrier family 22 member 3	− 62.3	7.8E − 04
00000118491.10	ZC2HC1B	Zinc finger C2HC-type containing 1B	− 64.3	5.6E − 05
00000138075.14	ABCG5	ATP binding cassette subfamily G member 5	− 67.3	4.4E − 04
00000273113.1		Novel transcript	− 69.0	3.9E − 04
00000271414.1		MYC associated factor X, pseudogene	− 89.3	3.3E − 06
00000255693.3	LINC02389	Long intergenic non-protein coding RNA 2389	− 98.2	8.3E − 07
00000163032.12	VSNL1	Visinin like 1	− 117.8	2.2E − 07
00000277518.3	MUC6	Mucin 6, oligomeric mucus/gel-forming	− 158.4	1.1E − 07

*Locus Tags preceeded by ENSG.

^#^pseudogene.

^carcinoembryonic antigen-related cell adhesion molecule 18.

### Novel contribution of GBS *sht* to evasion of phagocytosis and virulence

**Figure 4:**
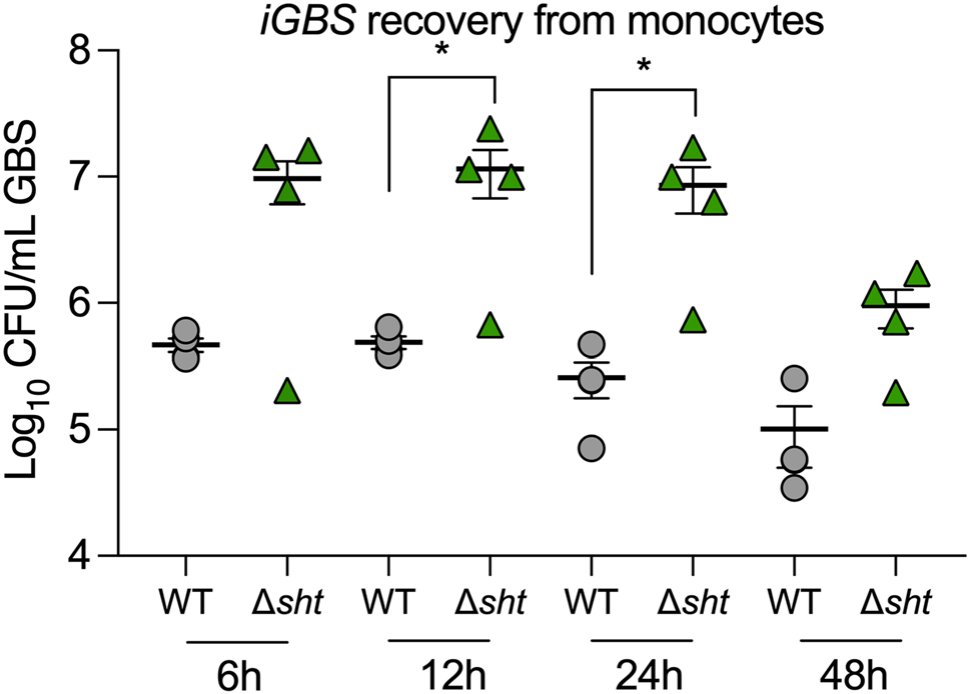
Novel contribution of *sht* in GBS to evasion of phagocytosis. Mutation in *sht* enhances the recovery of GBS from monocytes in comparison to WT (*n* = 4). U937 human monocytes were infected with a multiplicity of infection (MOI) of ~ 100 and extracellular bacteria were killed by antibiotic treatment, followed by quantification of *iGBS* at 6 h, 12 h, 24 h and 48 h post-infection. Bars show mean and SEM and unpaired *t* tests compared WT to Δ*sht* at each timepoint (*P ≤ 0.05).

Among the most significantly upregulated genes in *iGBS* was *sht* (sevenfold; Supplementary Dataset S1) encoding a Streptococcal Histidine Triad Protein. In initial qPCR assays targeting virulence factors of GBS, we noted *sht* was the most significantly upregulated of transcripts tested (Fig. 3). *sht* (Genbank Accession number ASZ01578.1) has been studied in other streptococci^35^ and encodes a surface expressed protein that is conserved in sequence and location among GBS strains^36^ and is regulated by the global virulence regulator *covR* in some, but not all GBS strains^37^. Sht supports Zn homeostasis in GBS and likely functions as an extracellular solute binding protein with affinity for Zn^38^. Interestingly, GBS has two versions of *sht* (termed *sht* and *shtII* in a prior study^38^), and we note that in our co-transcriptomic analysis, expression of *shtII* (Genbank Accession number ASZ02155.1) does not change. Thus, we examined *sht* to define whether this gene, as part of the GBS-monocyte co-transcriptome, might alter the host–pathogen interaction. We used antibiotic protection assays to compare the recovery of wild-type (WT) GBS and a targeted Δ*sht* mutant from monocytes over time at between 6 h and 48 h post-infection. We noted a major effect of deletion of *sht* on the relative recovery of GBS detecting significantly higher numbers of the Δ*sht* mutant compared to WT beginning at 12 h post-infection (Fig. 4). The Δ*sht* mutant was recovered in higher numbers compared to WT at all time points, however statistical significance was noted at only 12 h and 24 h (Fig. 4). Analysis of a Δ*sht* complementation strain with *in trans* expression of *sht* from a nisin-inducible ectopic promoter demonstrated restoration of counts of *iGBS* to levels equivalent to WT at 12 h post-infection (Supplementary Fig. S3).

**Figure 5:**
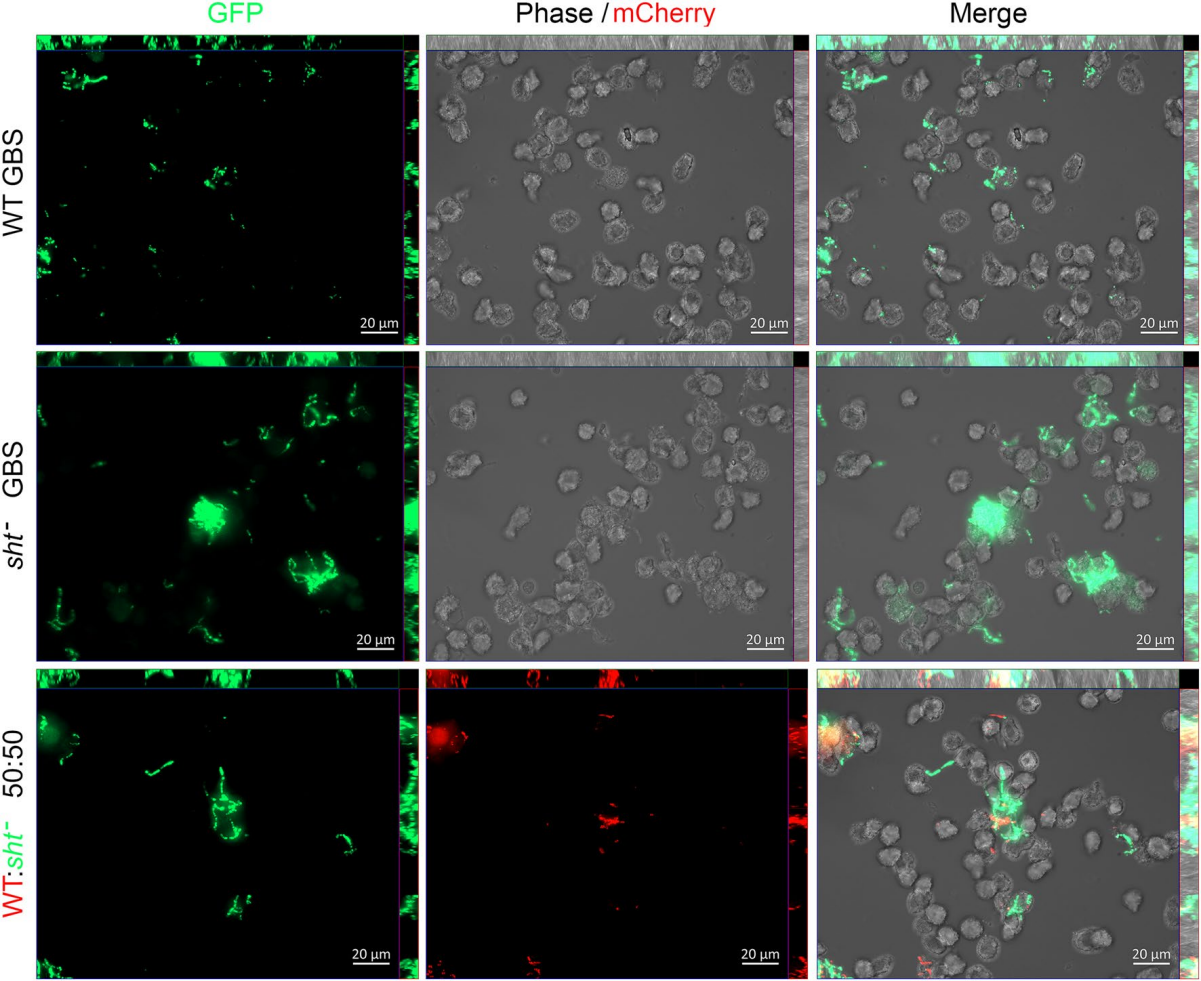
Visualization of the interaction between human monocytes, WT GBS and Δ*sht* GBS using fluorescence microscopy. The bacteria were tagged with either GFPmut3^24^ or mCherry^22^ and were used to infect monocytes in single infection assays (one strain of GBS) or mixed infection assays (both strains of GBS tagged with a different fluorophore, used in equal numbers). The images were acquired using a Zeiss AxioImager.M2 microscope and Zen Pro (version 2) software.

Analysis of GBS and monocytes using fluorescence microscopy confirmed more bacteria during infection with the Δ*sht* mutant, consistent with recovery data shown in Fig. 4, but not more monocytes affected by the infection. Single infection assays with either WT or Δ*sht* mutant tagged with GFPmut3^24^ showed the mutant in higher numbers with monocytes compared to the WT (Fig. 5). Mixed infection assays with equal numbers of WT-GFPmut3 and Δ*sht*-mCherry^22^ showed higher numbers of the mutant with monocytes compared to the WT (Fig. 5). Interestingly, we observed some monocytes with large numbers of bound GBS alongside other monocytes in the same field of view that had no visibly associated bacteria. Taken together, the results of antibiotic protection assays and microscopic analysis show that *sht* contributes to the ability GBS to evade phagocytosis and alters the survival of the bacteria inside the host cell.

**Figure 6:**
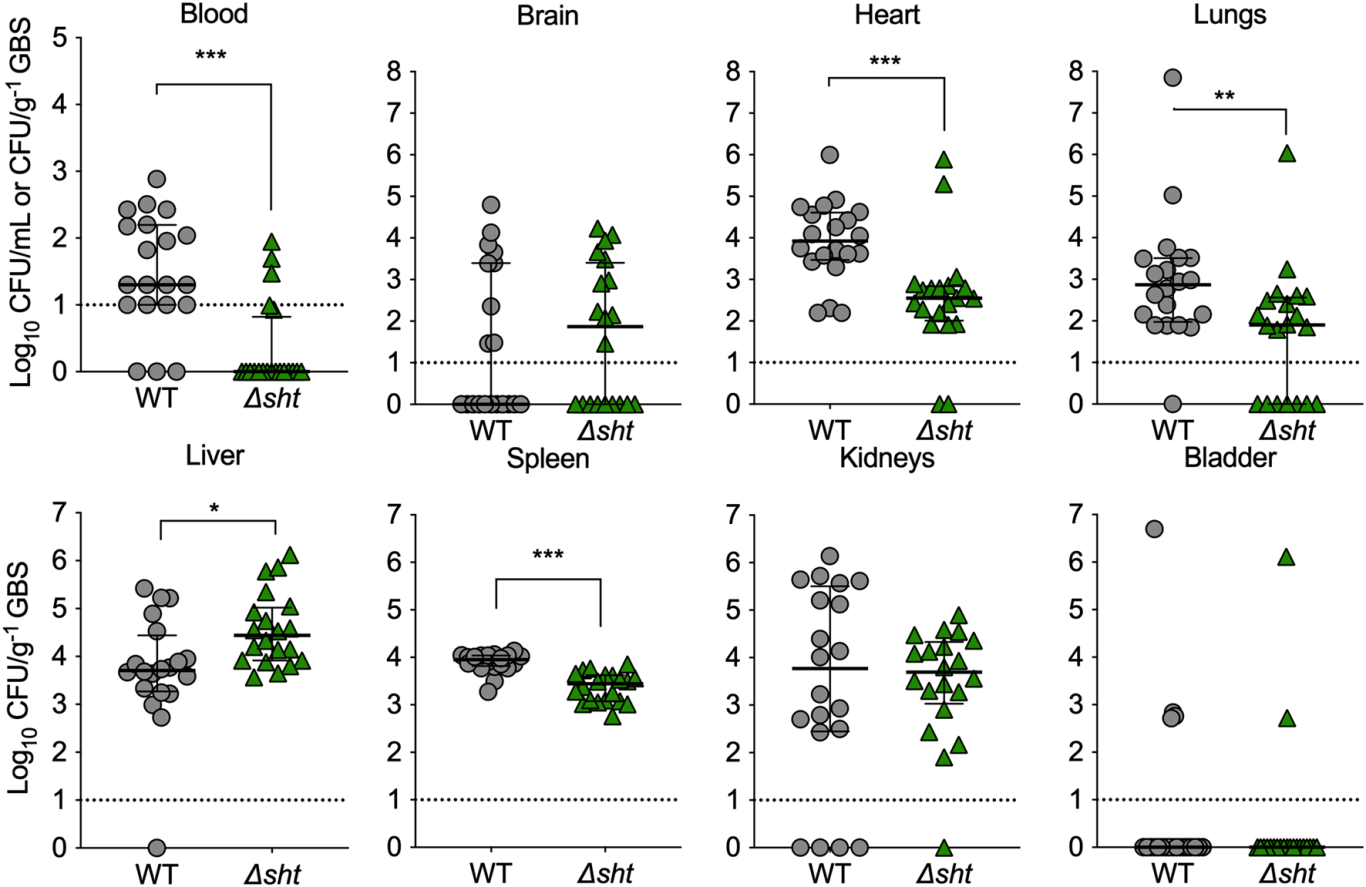
Attenuated Δ*sht* GBS compared to WT for survival in several tissues at 24 h after disseminated infection of mice (groups of *n* = 10 per independent experiment). Bars show median and IQR and represent two independent experiments analysed with Mann Whitney *U* tests. (*P ≤ 0.05; **P ≤ 0.01; ***P ≤ 0.001). Dotted lines represent limits of detection.

The contribution of *sht* to the host–pathogen interaction in the monocyte environment prompted us to examine its role in GBS virulence in vivo. Using a model of systemic infection in mice, remarkably, we found that *sht* supported survival of GBS in multiple organs, with significantly higher numbers of WT GBS recovered from the blood, heart, lungs and spleen of mice compared to the Δ*sht* mutant (Fig. 6). However, a significant contribution of *sht* was not observed in all the organs tested with no significant difference between the numbers of WT GBS and Δ*sht* mutant for the bladder and brain (Fig. 6). Taken together, these findings show that *sht* contributes to ability of GBS to respond to the intracellular niche, to evade phagocytosis, and to survive in the host during systemic disseminated infection in vivo.

## Discussion

This is the first study to explore GBS pathogenicity using dual RNA-seq^13,15^ in combination with the complete genomic information of the bacterium^28^ and models of host cellular and systemic infection that offers new insight into the host–pathogen interaction. Our combined application of these technologies to GBS-infected monocytes provides a new representation of how this pathogen adapts to the host cellular niche and adds definition to the transcriptional responses of the bacteria that effect virulence. The co-transcriptomic snapshot of the simultaneous gene responses of *iGBS* and monocytes highlights the engagement of genes that encode GBS virulence factors and innate host defence responses that are dominated by chemotactic and inflammatory signals. Overall, these findings provide new and comprehensive insight into the transcriptional responses that occur during GBS host–pathogen interactions.

The co-transcriptional response of *iGBS* inside human monocytes can be compared to prior studies to highlight some conserved and divergent responses. For example, upregulation of *lrgA* and *lrgB,* as well as *czcD*, and downregulation of the capsule (*cps*) gene cluster in its entirety, along with *pur* and *nrdEF* genes for nucleotide synthesis is consistent with responses of GBS exposed to whole human blood^10^. Other genes that encode virulence factors, including most of the *cyl* genes encoding a pathway for synthesis of the ornithine rhamnolipid hemolysin of GBS^39^, cell-surface anchored adhesins (*e.g., hvgA*, *fsbA*, *fsbB*, and *bspA*) and evasion factors (*e.g., scpB*, *cspA*, capsule *cps* loci) were dysregulated inside monocytes. The majority of these genes are regulated by the global virulence regulator CovRS^37,40–43^, suggesting the CovRS regulon may be important in *iGBS* survival, so the extent to which CovRS contributes to *iGBS* changes detected in this study requires further analysis.

We noted significant transcriptional dysregulation of other central cellular responses in *iGBS*, including genes for nucleotide synthesis pathways, metabolic enzymes, regulators and other factors. Several studies have highlighted the role of respiratory enzymes (*e.g., cydC*^44^) and nucleotide metabolism (*e.g., guaA*^45^) in GBS host–pathogen interactions. Notably, some of the responses detected in *iGBS* in this study are consistent with a recent analysis of GBS responses to Zn stress^22^. Specifically, 266/467 (57%) of gene responses identified in the prior study were also found to be significant in the current study. These encompass genes for Zn-efflux (*czcD*) and nickel transport (*nikABCD*), and genes for Cu homeostasis (*cutC*, *copZ*). It is interesting to speculate that some of the *iGBS* responses might reflect responses to monocyte defence based on accumulation of metal ions because Zn and Cu can be used by host cells to kill internalized bacteria^46,47^. Two recent studies that focused on Zn stress demonstrate a key role for *czcD* in resistance of GBS to Zn intoxication^22^ and a novel regulatory cross-talk mechanism mediated by CopY that supports GBS survival in conditions of Zn stress^48^. The extent to which metals are co-ordinated by monocytes against GBS will be important for future study.

The transcriptional changes in monocytes observed in this study following infection with GBS are broadly consistent with prior studies of innate host responses to GBS, which show that monocytes respond by upregulating the production of cytokines, chemokines and other factors that contribute to host defence^8^. Among the strongest responses was the gene for the CXC family chemokine IL8, recognised for its role in recruitment of neutrophils. Other responses that are consistent with previous studies of GBS and monocytes or monocyte-derived macrophages include strong induction of interferon gamma-induced protein 10 (CXCL10), and macrophage inflammatory protein-3alpha (CCL20), which have been related to strain-specific differences in GBS virulence^49^. Upregulation of CCR7 and CD80 in response to GBS has been associated with classically-activated, pro-inflammatory “M1” macrophage responses^50^; however *CD80* and other genes for surface glycoproteins (*e.g., CD36, CD86*) were found to be downregulated in our study along with upregulation of others, including *CD69* and *CD83*. This indicates that GBS triggers substantial remodelling of gene expression for surface markers and implies a distinct activation profile of monocytes comparing to binary M1 or M2 polarization states associated with macrophages. More broadly, these findings are consistent with known heterogeneity in surface marker expression among human monocyte subsets^51^. Finally, it is notable that few studies have investigated the mechanisms by which GBS enter into and/or are uptaken by human monocytes (as opposed to macrophages and neutrophils) and this could be an area for future investigation.

We elected to focus our functional analysis on a significant response of *iGBS* rather than monocytes in view of the dearth of knowledge of how GBS responds to the host cell niche. To achieve this, we tested an isogenic Δ*sht* targeted GBS mutant using monocyte and infection assays in mice to test whether GBS genes of the co-transcriptome can influence pathogenicity and shape the host–pathogen interaction. The *sht* gene was chosen because it was significantly up-regulated in *iGBS* cells, forms part of the CovRS regulon associated with virulence, and was recently shown to influence Zn uptake in GBS^38^. Like other human GBS isolates, strain 874391 used in the present study contains two Sht proteins with > 45% homology, encoded by *sht* and *shtII* which are co-located in the genome with other Zn-acquisition genes *lmb* and *adcAII*, respectively^38^. Both Sht and ShtII contain 5 distinct histidine motifs (HxxHxH) that, when mutated, impair the ability of GBS to grow in Zn-limited conditions^38^. Interestingly, *Streptococcus pneumoniae* has four Sht protein homologs (termed Pneumococcal histidine triad proteins, or Pht) which are also involved in Zn uptake^52,53^. Analysis of a Δ*sht* mutant revealed a significant effect of this gene in supporting evasion of phagocytosis and GBS survival in the host during systemic infection. In vitro, recovery of more Δ*sht* GBS as early as 6 h compared to WT GBS suggests that *sht* contributes to evading phagocytosis (*i.e.,* fewer WT GBS with Sht were recovered from the phagocytes); an alternative interpretation is that *sht* renders the WT bacteria more susceptible to killing by monocytes following internalization; however, the attenuated phenotype of Δ*sht* GBS observed in vivo, supports a virulence role for *sht* that would be consistent with bacterial evasion of phagocytosis. Interestingly, a previous study found that GBS Sht acts as a bacterial receptor for complement regulator, factor H (FH) and that binding of FH to GBS promotes resistance of the bacteria to innate immunity by facilitating inactivation of C3b and thereby, evasion of complement-mediated opsonization^54^. Given the predicted location of Sht at the cell surface, it would be of interest to examine the monocyte-GBS interaction in more detail, to determine rates of phagocytic uptake of the WT and Δ*sht* mutant, the extent to which Sht contributes to opsonin-independent versus -dependent recognition by monocyte cells in our model, and, the release of free Zn (or absence of) within monocytes during *iGBS* infection. Notably, it appears that expression of *sht*, *shtII* and *czcD* in monocytes differs markedly from currently accepted models of the regulation of Zn-responsive genes in GBS; *sht/shtII* are expressed in the absence of Zn^55^, conversely, *czcD* is expressed during conditions of excess Zn^22^. In the current study, both *sht* and *czcD* are up-regulated in *iGBS* (instead of being opposingly regulated), and *shtII* remains unchanged, implying alternative modes of regulation in GBS that are located inside host cells compared to in vitro grown bacteria. Notwithstanding, further examination of the contribution of *shtII* (or *czcD*) to *iGBS* survival is now warranted.

We utilized genome-sequenced GBS strain 874*,*391 as the basis to define the GBS-human monocyte co-transcriptome and validation approaches such as qPCR to confirm the co-transcriptional responses detected in this study. This approach represents a major advance on previous studies that have focused on either the host or the pathogen without a view of the simultaneous co-transcriptomic changes that occur during infection. Nonetheless, there are challenges to co-transcript studies with microbes, including the saturation of host RNA and the ability to recover sufficient, usable pathogen RNA. To address this, Bent et al*.*^56^ enriched for pathogen RNA using a novel capture-based technique. Ferreira-Machado et al*.*^57^ report ultrasonic treatment and centrifugation increased sequencing depth but noted degradation of RNA with increased yield. Humphrys et al*.*^58^ applied a method that used simultaneous depletion of both *Chlamydia* and human rRNA by affinity-based counter selection to enrich prokaryotic and eukaryotic RNA from infected cells. In our study, we optimized numerous conditions of infection, cell and RNA isolation, and used an extended sequencing depth to capture and quantify transcripts from GBS and host in sufficient quantity for robust analysis.

In the antibiotic protection assay for GBS-infected monocytes, we found that levels of FBS below 1% led to poor yield and quality of GBS RNA, and that a centrifugation step was essential to achieve sufficient yield of *iGBS*, similar to a prior study^59^. Centrifugation may impact transcript abundance^60,61^ but we unavoidably included centrifugation to increase GBS yield. Importantly, time-kill assays using antibiotics showed that the vast majority of extracellular GBS (> 99.99%) in the assay were dead at the time of cell harvest, with similar bactericidal effects (*i.e.,* 4 log_10_ reduction in CFU after 5 h in antibiotics versus no antibiotics) reported in prior studies^41,62^. Thus, the bulk of GBS that represent the source of RNA are *iGBS*, and extracellular bacteria are unlikely to contribute significantly to the RNA yield. Our microscopy analysis of fluorescent GBS bound to monocytes demonstrated some host cells to be associated with many GBS whereas other monocytes in the same culture were not clearly associated with bound bacteria. Detection of dissimilar numbers of bacteria bound to distinct host cells in the same culture vessel are not unusual^24^ but are largely unexplained.

In terms of RNA quality control, our approach to validate mixed population RNA as free of gDNA used PCRs to target GBS *spb1* and human *β-actin* and *gapdh*. Several studies have used various primers specific for *β-actin*^63–68^, and initially, we tested two previously published *β-actin* primer sets. However, non-specific amplicons led us to target *gapdh*^69,70^ instead. Ten primer pairs for *gapdh* were tested, and identified *gapdh* 7a primers as reliable and sensitive for this purpose.

A broader view of other host–pathogen co-transcriptomes highlights the unique nature of these responses and their implications for understanding the host–pathogen interaction. For example, in *S. pneumoniae* and human pleural mesothelial cells, the bacteria upregulate genes for adherence and metabolism^71^, and the host upregulates genes for stress responses and cell survival, but not for innate immune defence. Aprianto et al*.*^72^ reported that adherent *S. pneumoniae* can repress the expression of innate immune genes in epithelial cells^73^. Humphrys et al*.*^58^ revealed early Chlamydial transcriptional changes in epithelial cells, relating to dampening of host transcriptional responses. Mavromatis et al*.*^74^ used mouse macrophages with *E. coli* to show upregulation of immune signalling pathways. In human THP-1 cells infected with *Mycobacterium bovis*, upregulation of cholesterol biosynthesis pathways was linked to compensation for upregulation of cholesterol degradation pathways in the bacteria^75^.

The dysregulation of gene expression in the co-transcriptome of monocytes and *iGBS* underscores the uniqueness and impact of model systems (*e.g.,* host–pathogen cell types) used in dual RNA-seq. A summary view is that insights into host–pathogen interactions gained from dual RNA-seq are a function of each unique pathogen and host cell niche. Future study of the impact of elements of the co-transcriptome of GBS-infected human monocytes on infection will expand our insight into the virulence factors and host responses that shape these infections.

## Supplementary Information

Supplementary Information 1.

Supplementary Information 2.

## Data Availability

The dataset(s) supporting the conclusions of this article are available in the NCBI Gene Expression Omnibus repository, GEO accession GSE161013 in https://www.ncbi.nlm.nih.gov/geo/query/acc.cgi?acc=GSE161013.

## Abbreviations

*iGBS*: Intracellular GBS
MOI: Multiplicity of infection
GBS: Group B *Streptococcus*
RNA-seq: RNA-sequencing
Sht: Streptococcal histidine triad protein
