# Supplementary material for: Dual RNA sequencing of group B Streptococcus-infected human monocytes reveals new insights into host–pathogen interactions and bacterial evasion of phagocytosis
Source: Sci Rep. 2023 Feb 6;13:2137. doi: 10.1038/s41598-023-28117-x (PMC9902490; doi:10.1038/s41598-023-28117-x)
Supplement: Supplementary file 2 — Supplementary Information 2. [file 41598_2023_28117_MOESM2_ESM.docx]

**Dual RNA Sequencing of Group B *Streptococcus*-Infected Human Monocytes Reveals New Insights into Host-Pathogen Interactions and Bacterial Evasion of Phagocytosis**

Matthew J. Sullivan^1,3^ , Darren Prince^1^, Kelvin G. K. Goh^1^, Dean Gosling^1^, Lahiru Katupitiya^1^, Michael Crowley^2^, David K. Crossman^2^, and Glen C. Ulett^1#^

^1^School of Pharmacy and Medical Sciences, and ^2^Menzies Health Institute Queensland, Griffith University, Parklands, Australia 4222;

^2^Heflin Center for Genomic Sciences, University of Alabama at Birmingham, 1720 Second Ave. S., Birmingham, AL 35294-0024;

^3^School of Biological Sciences, University of East Anglia, Norwich, NR4 7TJ, UK

**Supplementary Materials.**

**Supplementary Table S1.**

**Supplementary Table S2.**

**Supplementary Table S3.**

**Supplementary Figure S1.**

**Supplementary Figure S2.**

**Supplementary Figure S3.**

**Supplementary Dataset S1. Complete list of transcriptional changes in co-transcriptome of GBS­-infected human monocytes.** Tab 1: A total of 1119 dysregulated transcripts were detected in *iGBS* with 745 upregulated and 374 down-regulated transcripts (±2-fold, P-adj <0.05). Tab 2: A total of 7587 dysregulated transcripts were detected in *iMon* with 7040 upregulated and 547 down-regulated transcripts (±2-fold, P-adj <0.05). Tab 3: Tab 4 Tab 5

**Supplementary Table S1.** Bacterial strains, plasmids and primers used in this study.

| **Bacterial Strains** | **Characteristics** | **Source** |
| --- | --- | --- |
| *E. coli* DH5α | *huA2 lac(*Δ*)U169 phoA glnV44 Φ80' lacZ(*Δ*)M15 gyrA96 recA1 relA1 endA1 thi-1 hsdR17* | Bethesda Research Labs |
| *S. agalactiae* 874391 | Genome sequenced Wild type, Sequence type-17, Serotype III strain, Vaginal isolate (Japan) | [^1^](#_ENREF_1)^,^[^2^](#_ENREF_2) |
| *S. agalactiae* GU2843 | 874391Δ*sht* mutant; Locus tag: CHF17_ RS06710; Cm | This work |
| *S. agalactiae* GU3178 | Complementation strain, GU2843 carrying plasmid pGU3173: 874391Δ*sht::sht* (Δ*sht* +C) | This work |
| *S. agalactiae* GU3180 | Empty vector Δ*sht* strain, GU2843 carrying plasmid pGU2356: 874391Δ*sht* (Δ*sht*^#^) | This work |
| *S. agalactiae* GU3181 | Empty vector WT strain, 874391 carrying plasmid pGU2356: 874391 (WT^#^) | This work |
| *S. agalactiae* GU2666 | *S. agalactiae* 874391 WT strain carrying plasmid pGU2664 (GFPmut3) | [^3^](#_ENREF_3) |
| *S. agalactiae* GU2667 | *S. agalactiae* 874391 WT strain carrying plasmid pGU2665 (mCherry) | [^4^](#_ENREF_4) |
| *S. agalactiae* GU3128 | Δ*sht* strain, GU2843 carrying plasmid pGU2664: GFPmut3Δ*sht* | This work |
| *S. agalactiae* GU3129 | Δ*sht* strain, GU2843 carrying plasmid pGU2665: mCherryΔ*sht* | This work |
| **Plasmids** | | |
| pHY304aad9 | *ori* (Ts); temperature-sensitive shuttle vector; Sp | [^5^](#_ENREF_5) |
| pLZ12 | *E. coli Streptococcus* shuttle vector, Cm | [^6^](#_ENREF_6) |
| pGU2812 | pHY304*aad9-*derivative *shtp*Δ construct; Sp, Cm | This work |
| pGU2356 | pMSP3545-derivative, NICE-inducible *Streptococcus* shuttle vector, Sp | [^5^](#_ENREF_5) |
| pGU3173 | pGU2356 containing 874391 *sht* directionally cloned into *Nco*I and *Bcu*I sites (2488 bp insert). | This work |
| pGU2664 | pDL278-derivative + GFPmut3, Sp | [^3^](#_ENREF_3) |
| pGU2665 | pDL278-derivative + mCherry, Sp | [^4^](#_ENREF_4) |
| **Primers** | **Oligonucleotide Sequence (5’-3’)** | **Application** |
| M13F | GTAAAACGACGGCCAG | Sequencing |
| M13R | CAGGAAACAGCTATGAC | Sequencing |
| 22up-1 | GATCCCGGGATTATCCGCTCAATGTCTCGAA | *sht* mutation |
| 23cat. 1-2 | CCAATTTTCGTTTGTTGAACTAAACCAAAAGAAGATCTCATTGTTA | *sht* mutation |
| 23cat. 1-5 | CTAATGTCACTAACCTGCCCCTAATAATCTCCTTTACTTCAACT | *sht* mutation |
| Lmb. 1-6 | GATCTCGAGACTTTATGACCCACATACCTGGAC | *sht* mutation |
| Htp_NcoF2 | GACCATGGTGAAGAAAACATATGGTTATATCG | *sht* complementation |
| Htp_BcuIR1 | GAACTAGTCAATGAGATCTTCTTTTGGTTAAGG | *sht* complementation |
| Htp-chk-f1 | TTGCCTAAATAAAGGAGCAATC | Sequencing |
| Htp-chk-R1 | AAGCTTGGGCAAGGGATCTA | Sequencing |
| dnaN-1F | CAACAAGAAAGCCGTCCAAT | qPCR |
| dnaN-1R | TCTGTCGCAACAGCCTTAAA | qPCR |
| hvgA-3F | TTTGGAATGGTCAGGAAGGA | qPCR |
| hvgA-3R | GCTGGTTTTGCAGCTTCTGT | qPCR |
| Rib-1F | CACCGACAACTTCAGGGACT | qPCR |
| Rib-1R | GCGATCCTAAAATGGCAAAA | qPCR |
| cpsE-1F | CACACGTGTCAGGGATCTTG | qPCR |
| cpsE-1R | ACAATTTCGACACGCATCAA | qPCR |
| scpB-1F | CTCTAGTGGCTGGTGCATTG | qPCR |
| scpB-1R | AGCTTTTGAGCCATTTGCTG | qPCR |
| cfb-1F | ACCCTAGATTGCGTGAGGAG | qPCR |
| cfb-1R | TGTCTTTGATGAAGCCACCA | qPCR |
| cylE-2F | AGGCGGCAATTATTTCACAC | qPCR |
| cylE-2R | ACTTGTCCATCAGGCTTCGT | qPCR |
| copA-1F | CCTTATCGCCAAACGTGATT | qPCR |
| copA-1R | ATTGCTAATTGGTGCCGTTC | qPCR |
| czcD-1F | TCAATATCTGGTCAATGGATGG | qPCR |
| czcD-1R | TAATGTTGGCAAATCGTTCG | qPCR |
| maeE-1F | CCACGTTGTTTCGAAATTGA | qPCR |
| maeE-1R | CGATTGCTGTACCATGTTGG | qPCR |
| 1233-1F (shtP) | TCCATGTCGTTCCGTATTCA | qPCR |
| 1233-1R (shtP) | CTCTTCATGCCCTGGCTTAG | qPCR |
| 029-GAPDH_F | CAAAAGGCGAAGAAGACTGAC | qPCR |
| 030-GAPDH_R | GGAACTTTGGCCATCTTGAC | qPCR |
| 033-IL1B_F | GGAGACTTGCCTGGTGAAAA | qPCR |
| 034-IL1B_R | CAGGGGTGGTTATTGCATCT | qPCR |
| 037-IL8_F | GGAGAACCTGAAGACCCTCA | qPCR |
| 038-IL8_R | TGCTCTTGTTTTCACAGGGA | qPCR |
| Human IL-10 Forward | CACTCCCAAAACCTGCTGAG | qPCR |
| Human IL-10 Reverse | TCTCTTCAGAAGTGCAAGGGTA | qPCR |
| 039-IL12A_F | AGATGGAATTTGGTCCACTGA | qPCR |
| 040-IL12A_R | GCAGGTGAAACGTCCAGAAT | qPCR |
| 045-TNF | TCCTGCATCCCCCATAGTTA | qPCR |
| 046-TNF | CTTCAGGAACAGCCACCAGT | qPCR |
| 049-LMO2_F | AACACTCACTCCACAACC | qPCR |
| 050-LMO2_R | TCCCCAAGTCTCTGTATCTA | qPCR |
| 051-MCP-1_F | TTTCCGCTCGTTAAAAAGGA | qPCR |
| 052-MCP-1_R | CACTTGCGTTGTTTGTGGAC | qPCR |

**Supplementary Table S2.** Prokaryotic-eukaryotic mixed RNA populations isolated from infected human monocytes containing intracellular (*iGBS*) and used for the dual RNA-seq.

| **Sample Designation** | **Combined Elutions (ng/µl)** | **Elution Volume (µl)** | **Total RNA (µg)** | **260/280** | **260/230** | **Amount for cDNA synthesis*** |
| --- | --- | --- | --- | --- | --- | --- |
| G175A | 21.9 | 60 | 1.3 | 2.10 | 0.99 | Not used |
| G175B | 12.2 | 60 | 0.7 | 2.02 | 0.75 | 172.8ng |
| G185A | 19.5 | 60 | 1.8 | 2.00 | 0.68 | 172.8ng |
| G185B | 17.8 | 60 | 1.1 | 2.12 | 0.81 | 172.8ng |
| G195A | 15.0 | 60 | 0.9 | 1.94 | 0.78 | 172.8ng |
| G195B | 17.3 | 60 | 1.0 | 2.09 | 0.46 | 172.8ng |
| G195C | 20.8 | 60 | 1.24 | 1.96 | 0.86 | 172.8ng |
| G195D | 17.0 | 60 | 1.0 | 1.98 | 0.75 | 172.8ng |
| G225A | 18.2 | 60 | 1.1 | 1.88 | 1.17 | 172.8ng |
| G225B | 7.2 | 60 | 0.43 | 1.87 | 0.44 | 172.8ng |
| G225C | 9.8 | 60 | 0.59 | 1.79 | 0.70 | 172.8ng |
| G225D | 8.8 | 60 | 0.53 | 1.77 | 0.42 | 172.8ng |
| M266 | 399.1 | 1000 | 399.1 | 2.07 | 2.17 | 5µg |
| M286 | 212.2 | 1000 | 212.2 | 2.02 | 2.11 | 5µg |
| M296 | 484.8 | 1000 | 484.8 | 2.05 | 2.18 | 5µg |
| M306 | 508.5 | 1000 | 508.5 | 2.06 | 2.18 | 5µg |
| U237A1 | 383.7 | 400 | 153.5 | 2.05 | 1.99 | 5µg |
| U237A2 | 393.5 | 400 | 158.2 | 2.04 | 2.08 | 5µg |
| U237A3 | 670.9 | 300 | 201.3 | 2.06 | 2.13 | 5µg |
| U247B1 | 352.6 | 400 | 141.0 | 2.04 | 2.18 | 5µg |
| U247B2 | 615.6 | 300 | 184.7 | 2.04 | 2.17 | 5µg |
| U247B3 | 625.8 | 300 | 187.7 | 2.05 | 2.11 | 5µg |

*For cDNA synthesis, RNA was extracted from four mixed (host+pathogen) samples (M266, M286, M296, M306), six U937 (host only) samples (U237A1, U237A2, U237A3, U247B1, U247B2, U247B3), and twelve GBS (pathogen only) samples (G175A, G175B, G185A, G185B, G195A, G195B, G195C, G195D, G225A, G225B, G225C, G225D). Dual RNA-seq was performed using n=4 for each of the three treatment groups.

**Supplementary Table S3.** Gene Ontology Over-Representation Analysis showing Biological Processes that are significantly up-regulated or down-regulated in *iMon* following infection with GBS. The total, up-regulated and down-regulated number of genes analysed in each GO category are shown with P-adjusted values indicating whether a pathway is statistically more over-represented in the uploaded dataset than expected by chance (Hypergeometric Distribution test with multiple comparisons Benjamini and Hochberg correction). Analysis used InnateDB (www.innateDB.com)

| **Biological Processes Over-represented in *iMon*** | | **Up-regulated genes** | | **Down-regulated genes** | |  |
| --- | --- | --- | --- | --- | --- | --- |
| **GO Term ID** | **GO Term Name** | **# genes** | **P-adj** | **# of genes** | **P-adj** | **# analysed** |
| GO:0006955 | immune response | 24 | <1.0E-5 | 54 | 1 | 221 |
| GO:0045087 | innate immune response | 55 | <1.0E-5 | 246 | 1 | 1027 |
| GO:0032496 | response to lipopolysaccharide | 17 | <1.0E-5 | 25 | 1 | 112 |
| GO:0006954 | inflammatory response | 22 | <1.0E-5 | 61 | 1 | 227 |
| GO:0009612 | response to mechanical stimulus | 10 | 0.000 | 7 | 1 | 48 |
| GO:0008285 | negative regulation of cell proliferation | 21 | 0.003 | 83 | 1 | 301 |
| GO:0001666 | response to hypoxia | 13 | 0.003 | 26 | 1 | 123 |
| GO:0010033 | response to organic substance | 10 | 0.003 | 17 | 1 | 72 |
| GO:0002467 | germinal center formation | 4 | 0.007 | 0 | 1 | 7 |
| GO:0006935 | chemotaxis | 10 | 0.008 | 19 | 1 | 82 |
| GO:0007050 | cell cycle arrest | 11 | 0.014 | 26 | 1 | 107 |
| GO:0007179 | transforming growth factor beta receptor signaling pathway | 11 | 0.016 | 25 | 1 | 110 |
| GO:0006915 | apoptotic process | 26 | 0.015 | 145 | 1 | 499 |
| GO:0006367 | transcription initiation from RNA polymerase II promoter | 13 | 0.020 | 43 | 1 | 158 |
| GO:0007267 | cell-cell signaling | 12 | 0.019 | 36 | 1 | 136 |
| GO:0014070 | response to organic cyclic compound | 10 | 0.022 | 28 | 1 | 97 |
| GO:0071222 | cellular response to lipopolysaccharide | 8 | 0.036 | 18 | 1 | 65 |
| GO:0043066 | negative regulation of apoptotic process | 21 | 0.039 | 114 | 1 | 385 |
| GO:2000352 | negative regulation of endothelial cell apoptotic process | 4 | 0.039 | 3 | 1 | 12 |
| GO:0035914 | skeletal muscle cell differentiation | 6 | 0.041 | 5 | 1 | 35 |
| GO:0042493 | response to drug | 15 | 0.048 | 71 | 1 | 229 |
| **Biological Processes Under-represented in *iMon*** | | **Up-regulated genes** | | **Down-regulated genes** | |  |
| **GO Term ID** | **GO Term Name** | **# genes** | **P-adj** | **# of genes** | **P-adj** | **# analysed** |
| GO:0006364 | rRNA processing | 2 | 1 | 59 | <1.0E-5 | 93 |
| GO:0008380 | RNA splicing | 1 | 1 | 111 | <1.0E-5 | 227 |
| GO:0000398 | mRNA splicing, via spliceosome | 0 | 1 | 87 | <1.0E-5 | 159 |
| GO:0008033 | tRNA processing | 0 | 1 | 38 | 3.0E-05 | 58 |
| GO:0000387 | spliceosomal snRNP assembly | 0 | 1 | 21 | 1.6E-04 | 26 |
| GO:0010467 | gene expression | 17 | 1 | 238 | 4.0E-04 | 615 |
| GO:0006270 | DNA replication initiation | 1 | 1 | 18 | 2.0E-03 | 23 |
| GO:0006260 | DNA replication | 2 | 1 | 70 | 2.0E-03 | 146 |
| GO:0034660 | ncRNA metabolic process | 0 | 1 | 17 | 2.0E-03 | 21 |
| GO:0000082 | G1/S transition of mitotic cell cycle | 3 | 1 | 67 | 3.0E-03 | 140 |
| GO:0006281 | DNA repair | 3 | 1 | 120 | 8.2E-03 | 291 |
| GO:0006521 | regulation of cellular amino acid metabolic process | 0 | 1 | 27 | 8.9E-03 | 44 |
| GO:0006271 | DNA strand elongation involved in DNA replication | 0 | 1 | 21 | 8.9E-03 | 31 |
| GO:0006446 | regulation of translational initiation | 0 | 1 | 23 | 1.3E-02 | 36 |
| GO:0006200 | ATP catabolic process | 1 | 1 | 100 | 1.6E-02 | 239 |
| GO:0001731 | formation of translation preinitiation complex | 0 | 1 | 13 | 1.7E-02 | 16 |
| GO:0090501 | RNA phosphodiester bond hydrolysis | 0 | 1 | 15 | 2.0E-02 | 20 |
| GO:0015684 | ferrous iron transport | 0 | 1 | 11 | 2.7E-02 | 13 |
| GO:0000375 | RNA splicing, via transesterification reactions | 0 | 1 | 17 | 3.3E-02 | 25 |
| GO:0006369 | termination of RNA polymerase II transcription | 0 | 1 | 26 | 4.9E-02 | 46 |

**Supplementay Figure S1
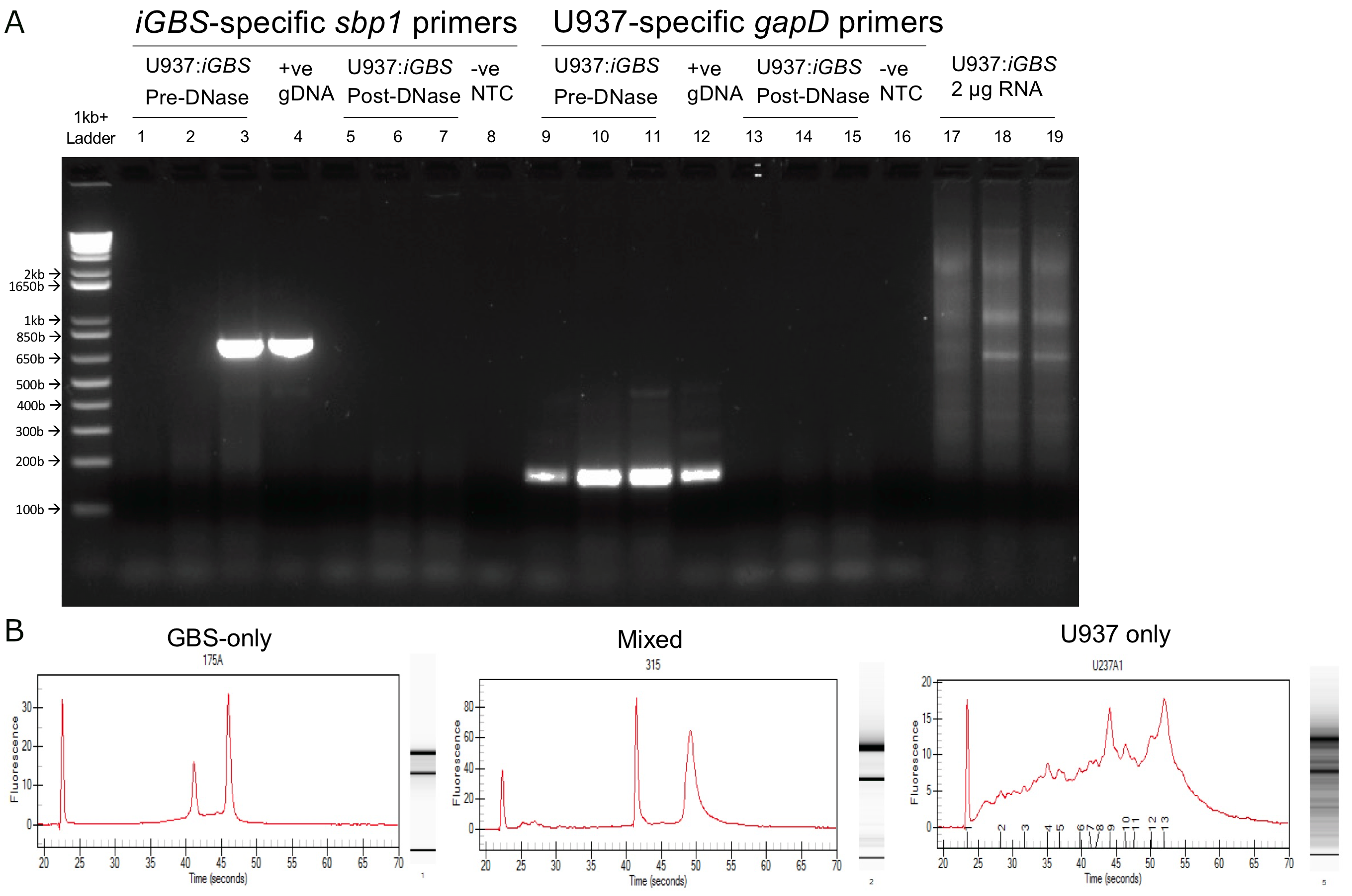
**

**Supplementay Figure S1. Simultaneous recovery of gDNA-free RNA from *iGBS* and infected monocytes.** PCR assay using primers for *spb1* of GBS and human *gapdh* for confirmation of RNA isolated from monocytes containing *iGBS* as free from gDNA (A). The prokaryotic-eukaryotic mixed RNA populations (U937:*iGBS*) were treated with DNase using Turbo DNA-free (Invitrogen), and compared with gDNA isolated from independent pure cultures of GBS or monocytes as positive controls (+ve). NTC, no template (-ve) control. Analysis of RNA from GBS-infected monocytes or control RNA samples by automated electrophoresis using Experion Prokaryote or Eukaryote RNA StdSens Chips in an Automated Electrophoresis Station (Bio-Rad) illustrating RNA of sufficient quality for sequencing (B). Electropherograms and virtual gel images (inset) from a single sample for each group (GBS-only, Mixed, U937-only) is shown, representative of 4 independent replicates for each condition. Cropped virtual gel images (B) are displayed with full-length virtual gels included in Supplementary Information as Supplementay Figure S2.

**Supplementay Figure S2**

**
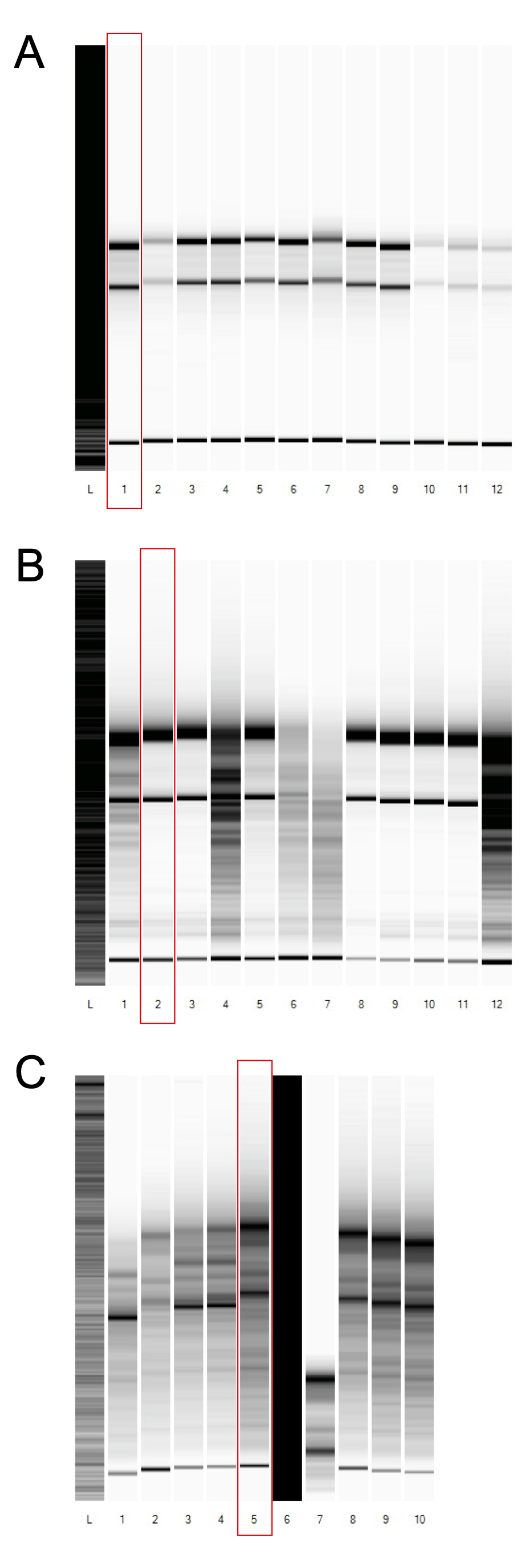
**

**Supplementay Figure S2.** Full-length virtual gel images for analysis of RNA from GBS-infected monocytes and control RNA samples. Electrophoresis was performed using Experion Prokaryote or Eukaryote RNA StdSens Chips in an Automated Electrophoresis Station (Bio-Rad). A single sample for each group (A, GBS-only [lane 1]; B, Mixed [lane 2], U937-only [lane 5]) is shown (highlighted in red boxes), representative of 4 independent replicates for each condition, and relates to cropped virtual gel images shown in Figure 2B.

**Supplementay Figure S3**


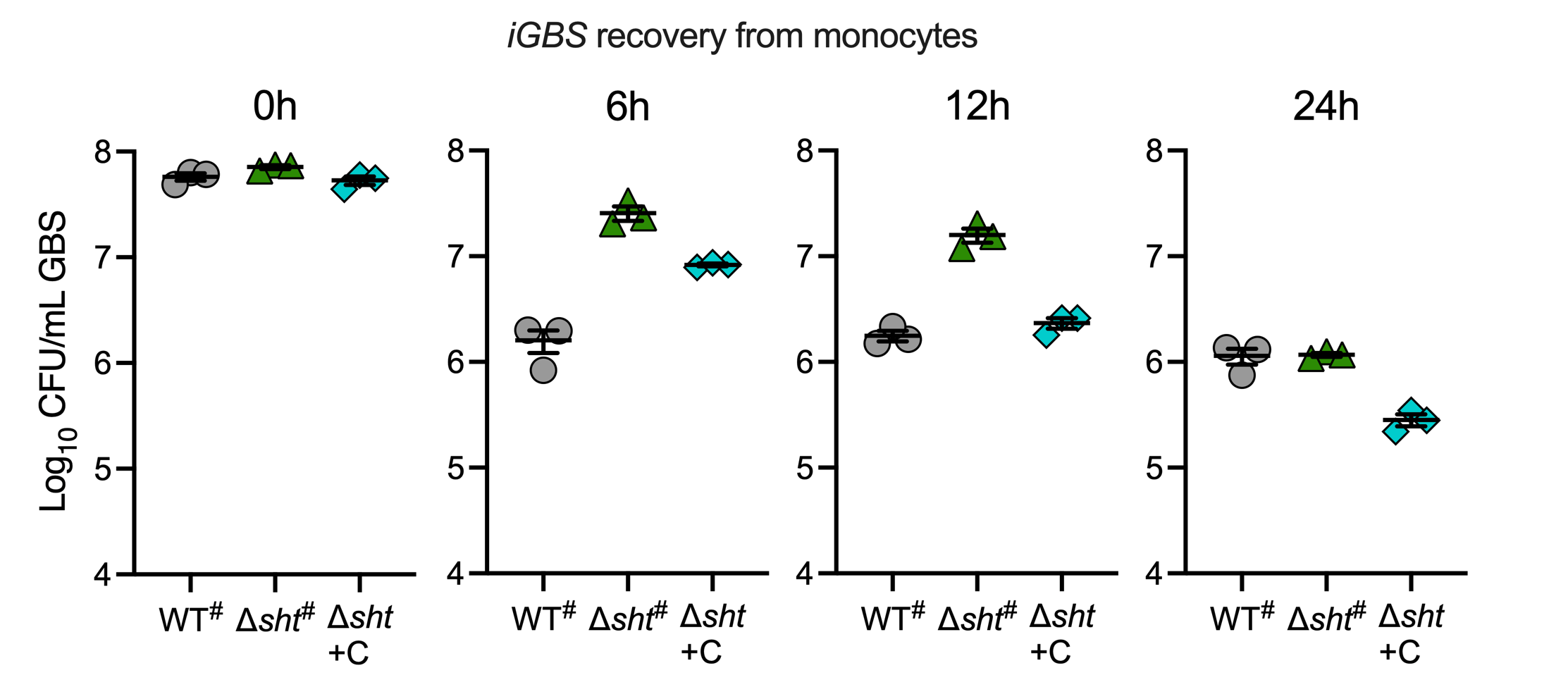


**Supplementary Figure S3. Complementation of GBS *sht* mutation in monocyte infection assay.** U937 human monocytes were infected with GBS at a multiplicity of infection (MOI) of ~100 and extracellular bacteria were killed by antibiotic treatment, followed by quantification of *iGBS* at 0h, 6h, 12h, and 24h post-infection. Both the WT GBS strain and the Δ*sht* mutant strain contained empty vector pGU2356 (indicated with ^#^ ; derived from pMSP3545; Sp^R^). Complementation of the *sht* mutation involved cloning *sht* downstream of the nisin-inducible pNisA promoter in pGU2356 to form pGU3173 (Δ*sht* + C). WT^#^, Δ*sht*^#^ and Δ*sht* +C strains were pre-grown overnight in THB + Spec and 20IU/mL nisin to induce expression of *sht* in the complemented strain and enable controlled comparisons to the WT^#^ and Δ*sht*^#^ strains in identical growth conditions. To maintain *sht* expression during the antibiotic protection assay, Sp and nisin (20IU/mL) were added together with other antibiotics. Bars show mean and SEM from n=3 technical replicates.

**Supplementary References**

1 Takahashi, S. *et al.* Role of C5a-ase in group B streptococcal resistance to opsonophagocytic killing. *Infect. Immun.* **63**, 4764-4769 (1995).

2 Sullivan, M. J. *et al.* Complete Genome Sequence of Serotype III *Streptococcus agalactiae* Sequence Type 17 Strain 874391. *Genome Announcements* **5**, doi:10.1128/genomeA.01107-17 (2017).

3 Sullivan, M. J. & Ulett, G. C. Stable Expression of Modified Green Fluorescent Protein in Group B Streptococci To Enable Visualization in Experimental Systems. *Appl. Environ. Microbiol.* **84**, doi:10.1128/AEM.01262-18 (2018).

4 Sullivan, M. J., Goh, K. G. K. & Ulett, G. C. Cellular Management of Zinc in Group B Streptococcus Supports Bacterial Resistance against Metal Intoxication and Promotes Disseminated Infection. *mSphere* **6**, doi:10.1128/mSphere.00105-21 (2021).

5 Ipe, D. S. *et al.* Discovery and Characterization of Human-Urine Utilization by Asymptomatic-Bacteriuria-Causing *Streptococcus agalactiae*. *Infect. Immun.* **84**, 307-319, doi:10.1128/IAI.00938-15 (2015).

6 Pritzlaff, C. A. *et al.* Genetic basis for the beta-haemolytic/cytolytic activity of group B Streptococcus. *Mol. Microbiol.* **39**, 236-247 (2001).
